# IR spectroscopic characterization of [M,C,2H]^+^ (M = Ru and Rh) products formed by reacting 4d transition metal cations with oxirane: Spectroscopic evidence for multireference character in RhCH_2_^+^†

**DOI:** 10.1039/d4cp00012a

**Published:** 2024-03-15

**Authors:** Frank J. Wensink, Corry E. Smink, Brandon C. Stevenson, Ryan P. Steele, Joost M. Bakker, P. B. Armentrout

**Affiliations:** a Radboud University, Institute for Molecules and Materials, HFML-FELIX Toernooiveld 7 6525 ED Nijmegen The Netherlands joost.bakker@ru.nl; b Department of Chemistry, University of Utah 315 South 1400 East Salt Lake City Utah 84112 USA peter.armentrout@chem.utah.edu

## Abstract

A combination of infrared multiple-photon dissociation (IRMPD) action spectroscopy and quantum chemical calculations was employed to investigate the [M,C,2H]^+^ (M = Ru and Rh) species. These ions were formed by reacting laser ablated M^+^ ions with oxirane (ethylene oxide, c-C_2_H_4_O) in a room-temperature ion trap. IRMPD spectra for the Ru species exhibit one major band and two side bands, whereas spectra for the Rh species contain more distinct bands. Comparison with density functional theory (DFT), coupled-cluster (CCSD), and equation-of-motion spin–flip CCSD (EOM-SF-CCSD) calculations allows assignment of the [M,C,2H]^+^ structures. For the spectrum of [Ru,C,2H]^+^, a combination of HRuCH^+^ and RuCH_2_^+^ structures reproduces the observed spectrum at all levels of theory. The well-resolved spectrum of [Rh,C,2H]^+^ could not be assigned unambiguously to any calculated structure using DFT approaches. The EOM-SF-CCSD calculations showed that the ground-state surface has multireference electronic character, and symmetric carbenes in both the ^1^A_1_ and ^3^A_2_ states are needed to reproduce the observed spectrum.

## Abstract 2

A combination of infrared multiple-photon dissociation (IRMPD) action spectroscopy and quantum chemical calculations was employed to investigate the [M,C,2H]^+^ (M = Ru and Rh) species.

## Graphical abstract

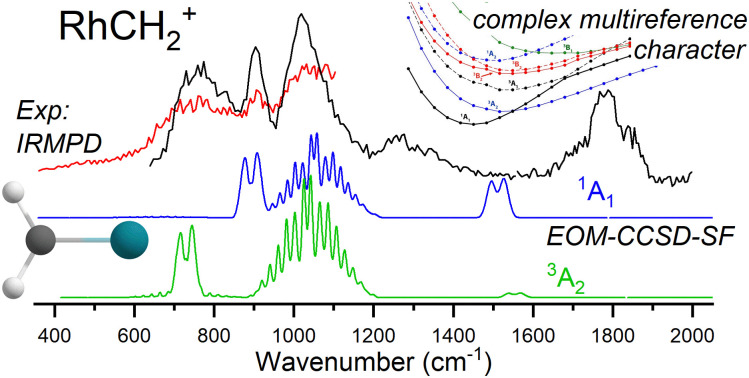

## Introduction

The functionalization of C–H bonds is a longstanding topic in chemistry.^1,2^ Functionalization reactions start with activation, which is a bottleneck for energy-efficient and selective functionalization processes involving C–H bonds because of their typically high bond energies. As the major component of natural gas and the simplest alkane molecule, methane is the most commonly studied alkane, often with the objective to investigate how to selectively convert methane into liquid fuels like methanol that remain energy rich but are more easily transported. Methane activation catalysis is currently neither specific nor energy efficient enough for direct utilization of methane. Rather, current schemes rely on thermal cracking, followed by synthetic pathways to more valuable chemicals. For more efficient, direct utilization of methane, it is imperative to develop better C–H bond activation catalysts.

Rational design of more active and selective catalyst materials requires a detailed understanding of the interaction between potential catalyst materials and substrates. One way to obtain this information for methane is to study its interactions with model systems for the active site.^3–5^ To obtain the most detailed information, the reaction can be isolated in the gas phase, which removes the perturbing effects of a support or solvent. This approach enables studies to focus on the intrinsic interactions. Once such interactions are understood in detail, support, solvent, and pressure effects can be considered.

Previously, studies have shown that methane is activated at room temperature by the third-row (5d) transition metal (TM) cations, Ta^+^, W^+^, Os^+^, Ir^+^, and Pt^+^, leading to dehydrogenation and the formation of [M,C,2H]^+^ products.^6–17^ Infrared (IR) spectroscopic characterization of [M,C,2H]^+^ products showed that [Pt,C,2H]^+^ has a PtCH_2_^+^ carbene structure with *C*_2v_ symmetry, whereas [Ta,C,2H]^+^ and [W,C,2H]^+^ are carbene structures that are distorted by agostic interactions.^18–21^ In contrast, [Os,C,2H]^+^ and [Ir,C,2H]^+^ have a hydrido metal carbyne HMCH^+^ structure, and for Ir^+^, experimental evidence was also found for the co-existence of a higher-energy symmetric IrCH_2_^+^ carbene structure.^18–20,22^ The observation of the HIrCH^+^ structure demonstrated the need for spectroscopic characterization, because the same structure had been discarded earlier on energetic grounds.^23^

In contrast to third-row TM cations, none of the second-row (4d) TM cations dehydrogenates methane exothermically,^15,24–37^ although the reaction with Zr^+^ is only slightly endothermic.^38^ To understand the underlying reasons why reactions of the lighter TM cations are endothermic, it is of interest to characterize the structures of [M,C,2H]^+^ products where M is a second-row TM. Because Os^+^ and Ir^+^ were shown to be the most reactive ions for methane dehydrogenation and also show the most diversity in their structures,^13,16–19,22^ the present study focuses on the bonding nature and structures of [M,C,2H]^+^ with their second-row congeners: the elements of groups 8 and 9, M = Ru and Rh. Earlier studies investigated the M^+^–CH_2_ bond strengths for first-row TMs and found that the bond dissociation energy (BDE, *D*_0_) decreases with an increase in the promotion energy (required for promoting a metal atom in its ground state to an electron configuration where there is one electron in the valence *s* orbital that is spin-decoupled from the remaining metal d electrons) of the atomic metal cation.^39^ It seems likely that a parallel trend is followed for the second-row TMs considered here, although this has not been quantified. The value of *D*_0_(Ru^+^–CH_2_) has been determined to be 3.57 ± 0.05 eV,^37^ such that formation of RuCH_2_^+^ and H_2_ from Ru^+^ and CH_4_ requires 1.17 ± 0.05 eV given *D*_0_(CH_2_–H_2_) = 4.743 ± 0.001 eV.^40^ Analogously, *D*_0_(Rh^+^–CH_2_) has been determined as 3.69 ± 0.08 eV in guided ion beam experiments^41^ and as 4.08 ± 0.22 eV by ion–molecule bracketing reactions.^42^ Thus, formation of RhCH_2_^+^ and H_2_ from Rh^+^ and CH_4_ requires an energy of 1.03 ± 0.08 (0.67 ± 0.22) eV.^32^

Elsewhere, a B3LYP potential energy surface (PES) for the reaction between Ru^+^ and CH_4_ identified the first step as physisorption with an exothermicity of 0.78 eV.^37^ This energy can be used to transfer one hydrogen atom from methane to Ru^+^, forming H–Ru^+^–CH_3_, which still lies 0.18 eV below the energy of the reactants. A further step toward (H_2_)RuCH_2_^+^ requires the crossing of endothermic energy barriers and a spin flip from quartet to doublet. Formation of the doublet-spin (H_2_)RuCH_2_^+^ intermediate is still exothermic by 0.09 eV, but loss of H_2_ requires over 1 eV, such that the overall dehydrogenation reaction to form RuCH_2_^+^ (^2^A_2_) + H_2_ is calculated to be endothermic by 1.08 eV. Energetically more favorable are the formation of the doublet-spin HRuCH^+^ (+0.97 eV) and quartet-spin RuCH_2_^+^ (+0.94 eV) products. Formation of the former has no barriers exceeding the product energy, whereas progress along the quartet-spin surface requires passing over a tight-transition state barrier of 1.03 eV.^37^

For RhCH_2_^+^, multireference – single double configuration interaction – complete active space self-consistent field (MR-SDCI-CASSCF)^43^ and B3LYP^44^ calculations have identified a ^1^A_1_-state carbene as lowest in energy, followed by a ^3^A_2_ state lying 0.20 and 0.19 eV, respectively, higher in energy. (The relative energies of higher-lying states identified in the former study are included in Table S1, ESI†). Again, the only intermediate that can be formed exothermically on the PES of Rh^+^(^3^F) + CH_4_ is the physisorption step;^43,44^ however, the CASSCF calculations found a barrier of about 0.4 eV for the reverse RhCH_2_^+^ + H_2_ reaction, whereas the later B3LYP calculations found no barrier. The latter result agrees with experiments for both the forward and reverse reactions.^32,42^

As described above, none of the metal cations we study here react exothermally with methane.^26,31,32,37^ Therefore, in order to form the [M,C,2H]^+^ species of interest, we react the corresponding M^+^ ions with oxirane (ethylene oxide, c-C_2_H_4_O). Oxirane readily reacts with many TM cations because of its ring strain and because [M,C,2H]^+^ formation is accompanied by the stable CH_2_O neutral product. Thus, extraction of CH_2_ from oxirane requires much less energy than from methane, *D*_0_(CH_2_–CH_2_O) = 3.375 ± 0.004 eV, compared to the *D*_0_(CH_2_–H_2_) = 4.743 ± 0.001 eV already mentioned.^40^ In contrast to the endothermic reactions with methane, the thermochemistry noted above indicates that formation of MCH_2_^+^ + CH_2_O in the reaction of M^+^ with oxirane is exothermic by 0.20 ± 0.05 and 0.32 ± 0.08 eV for M = Ru and Rh, respectively. Although it has been shown that laser-ablated Rh^+^ reacts at thermal energies with oxirane to yield RhCH_2_^+^,^42^ product distributions for the reactions of Ru^+^ and Rh^+^ with c-C_2_H_4_O have not been published previously.

In the present work, we probe the structures of the [M,C,2H]^+^ product ions for M = Ru and Rh using a combination of IR multiple photon dissociation (IRMPD) action spectroscopy and DFT or coupled cluster with single and double excitations (CCSD) calculations. IRMPD spectra are recorded using the same Fourier transform ion cyclotron resonance (FTICR) mass spectrometer recently used to show that the reaction of Pt^+^ with two methane molecules leads to the formation of a Pt^+^(ethene) complex following two dehydrogenations and C–C coupling.^21^ In that same work, we demonstrated that the spectrum of [Pt,C,2H]^+^ recorded with this instrument is consistent with that reported previously,^18^ where [Pt,C,2H]^+^ was formed in a molecular beam environment without mass-isolation prior to irradiation. Earlier attempts to record IRMPD spectra of [M,C,2H]^+^ with M = Ru were unsuccessful in the molecular beam apparatus because the ion intensities were insufficient. Here, we take advantage of the possibility to react TM^+^ with c-C_2_H_4_O over longer times and to mass-isolate the formed [M,C,2H]^+^ species in order to obtain the desired spectra.

Critically, these small ions also form benchmark systems for the accuracy of quantum chemistry methods. Previously, we found that relatively standard DFT methods proved sufficient to accurately predict the IR fingerprints, both in terms of frequencies and IR intensities that are necessary for assigning the experimental IR spectra of [M,C,2H]^+^ species with 5d TM elements.^18–22^ In contrast, in a parallel publication investigating the IR spectra of FeCH_2_^+^ and CoCH_2_^+^,^45^ 3d congeners of the species currently under investigation, we found that a large variety of DFT methods provide a significant underestimation of the IR absorption intensity for the M–C stretch vibration. We provided computational evidence suggesting that this relates to inaccurate charge separation of these DFT methods. One motivation of the present work is therefore to assess whether DFT treatments of this mode are as poor as they were for 3d elements, or whether they are accurate enough, like for the 5d elements studied earlier. We therefore also employ higher-level single- and multireference methods to interpret the observed spectra. One previous computational study,^43^ based on CASSCF and MR-SDCI-CASSCF methods, noted only modest multireference contributions to the ground-state wavefunction of RhCH_2_^+^. The same analyses were unable to reproduce the experimentally observed Rh^+^–CH_2_ binding energy and yielded relative electronic state energies that differ appreciably from the ones computed herein. We find that adequately treating the multireference character, while employing modern core potentials and basis sets, is needed to properly reproduce the observed spectrum of RhCH_2_^+^. In the case of [Ru,C,2H]^+^, multi-reference character does not appear to be relevant, but here contributions from multiple structures and states, whose energies vary with the level of theory, are required to match the experimental spectrum.

## Methods

### Experimental

Atomic metal (Ru, Rh) cations were produced in a laser vaporization source.^46,47^ In this source, a solid metal target disk was irradiated by a frequency doubled Nd:YAG laser at 30 Hz in the presence of helium gas injected by a pulsed valve. The helium cooled the metal ions through collisions and guided them toward the end of the source, where the gas mixture adiabatically expanded into vacuum, thereby further cooling the ions. They were then transferred, *via* a quadrupole mass filter in guidance mode, to a quadrupole ion trap with rectangular electrodes where they were trapped by dissipating the kinetic energy with 5 × 10^−4^ mbar of argon buffer gas. In the trap, the metal ions reacted with oxirane at a partial pressure of 1 × 10^−6^ mbar, which was mixed into the argon. After accumulating ions for approximately 400 ms, the voltage on the exit electrode of the ion trap was lowered. This released the ions from the ion trap after which they were guided into the cavity of the Free Electron Laser for IntraCavity Experiments (FELICE). Here, the ions were trapped in one of the cells of the FTICR mass spectrometer integrated within the laser cavity. This FTICR has four cells, with the first (cell 1) located in the laser focus and the fourth 30 cm from the focus, thereby lowering the photon fluence by a factor of 14. After ion capture, all unwanted masses were ejected from the FTICR cell using a combination of radio frequency (rf) excitation pulses (chirped and single-frequency),^48^ so that only the species of interest were irradiated by IR light produced by FELICE. Resonant absorption of IR photons increased the internal energy of the ions and once the fragmentation threshold was overcome, the ions dissociated, which indicates the presence of a vibrational band at the resonant IR frequency. The IRMPD spectrum was obtained by recording the intensities of precursor (*I*_p_) and fragment (*I*_frag_) ions present in the FTICR cell after irradiation and is expressed as the fragmentation yield *Y*:
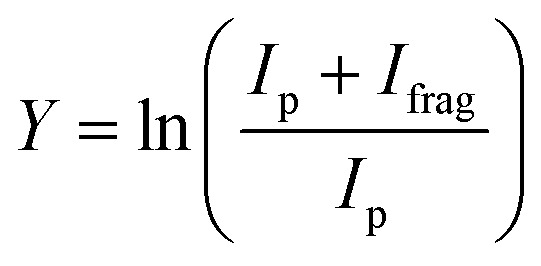


The IR light of FELICE is tunable in the 100–2000 cm^−1^ spectral range, although for this work only the 300–2000 cm^−1^ spectral range was used. The spectral bandwidth was near transform limited and, during these measurements, the full-width at half-maximum (FWHM) was approximately 0.7% of the central frequency. FELICE produced micropulses at 1 GHz, and the macropulses lasted between 6–10 μs and were repeated at 5 or 10 Hz. All spectra were recorded using a single macropulse with the ions in FTICR cell 4 (low fluence) unless specifically indicated otherwise. FELICE macropulse energies used ranged from 0.3 J at lower frequencies to 0.5 J at higher frequencies, corresponding to macropulse fluences ranging from 0.9 to 6.6 J cm^−2^ in FTICR cell 4.

### Computational

The DFT calculations were done using the Gaussian 16 software package,^49^ where all molecular structures, closed- and open-shell, were optimized using the unrestricted B3LYP hybrid functional and the def2-TZVPPD basis set.^50–52^ This combination was used because the M^+^–CH_2_ BDEs calculated for the metals explored here matched the experimental values relatively well: *D*_0_(Ru^+^–CH_2_) = 3.73 eV (experimental 3.57 ± 0.05 eV) and *D*_0_(Rh^+^–CH_2_) = 3.60 eV (experimental 3.69 ± 0.08 eV). All electrons were treated explicitly for H and C, whereas an effective core potential (ECP) of 28 electrons (small core) was used for Ru and Rh with the 4s, 4p, 5s, and 4d orbitals treated explicitly by the basis set. To ascertain true minima and for comparison with the experimental spectra, harmonic vibrational frequencies were calculated. All relative energy values provided below include harmonic zero-point vibrational energies. For each vibrational mode, rovibrational envelopes were simulated assuming pure a-, b-, or c-type transitions on the basis of moments obtained from the computations.^53^ For comparison with experiment, the simulated rotational substructures were shifted to the unscaled, calculated vibrational frequencies and the individual rotational transition intensities multiplied by the vibrational transition intensities. (For these calculations, no scaling factors were used to compare the theoretical spectra with experiment, which is unusual for these types of systems.^18–22,54–56^ Nevertheless, such scaling factors were not found to improve the agreement between theory and experiment and therefore are not used throughout this work except as noted.) The resulting rovibrational transition lines were subsequently convoluted using a Gaussian line shape function with a FWHM of 0.9% of the central frequency, mimicking the FELICE spectral bandwidth. It should be noted that because all calculated spectra correspond to absorption of a single IR photon, the predicted intensities can differ from the experimental multiple-photon spectra.

Additional geometry and frequency calculations were performed at the second-order Möller–Plesset, MP2,^57^ and coupled-cluster with single and double excitations, CCSD,^58^ levels using the same def2-TZVPPD basis set. These calculations were followed by single-point calculations with added perturbative triple excitations, *i.e.*, at the CCSD(T)/def2-TZVPPD level of theory.

Because of the large degree of spin contamination initially observed in DFT (and even CCSD) computations of the singlet [Rh,C,2H]^+^ and doublet [Ru,C,2H]^+^ species, additional calculations were performed with the equation-of-motion spin–flip^59,60^ CCSD (EOM-SF-CCSD) level of theory using the cc-pVTZ-PP basis set^61^ and the ECP28MDF pseudopotential^61^ on Ru/Rh and the cc-pVTZ basis set^62^ on C and H. To clarify relative energies, perturbative triples were added *via* single-point computations with the EOM-SF-CCSD using the perturbative Fock triples correction (fT)^63^ on optimized structures. The Q-Chem software package was utilized for the MP2, CCSD, and EOM computations, and the 4s and 4p electrons on Ru/Rh and the 1s electrons on C were frozen for the electron-correlation calculations. The ground triplet state was used as the spin–flip reference for [Rh,C,2H]^+^. For [Ru,C,2H]^+^, a peculiar symmetry restriction required special treatment: the effectively doubly degenerate ground quartet states of B_1_ and B_2_ symmetry led to independent (and also nearly degenerate) manifolds of spin–flip states. Therefore, both quartets were used as spin–flip references, and properties of both manifolds will be presented herein. In the asymmetric HRuCH^+^ configuration, the lowest ^4^A′′ state was used as the spin–flip reference. The structures of at least the two lowest-energy electronic states of each spin–flip transition symmetry were first optimized with the analogous cc-pVDZ-PP basis set, using a series of disparate starting structures. Stable structures were then re-optimized with the cc-pVTZ-PP basis, and harmonic zero-point corrections and spectra were computed. Total electronic densities, spin densities, 〈*S*^2^〉 values, and Mulliken spin populations and charges were computed with the resulting EOM-SF-CCSD wavefunctions. Constrained potential energy curves along the metal–carbene coordinate were also computed by optimizing the structure of the lowest-energy electronic state at a series of Rh–C(A_1_)/Ru–C(B_1_/B_2_) distances between 1.5 and 2.5 Å. The energies of all other electronic states were computed as vertical single-points from these structures. Here, adequate comparison of the predicted IR spectrum for [Rh,C,2H]^+^, but not [Ru,C,2H]^+^, with experiment required the use of frequency scaling factors, which are detailed below.

## Results & discussion

Reactions between M^+^ (M = Ru, Rh) and oxirane resulted in the ionic products MC^+^, [M,C,2H]^+^, [M,O,2H]^+^ for both M, MO^+^ and [M,2C,2H]^+^ for M = Ru, and MH^+^, [M,C,3H]^+^, [M,C,O]^+^, and [M,C,O,H]^+^ for M = Rh. These product mass distributions are shown in Fig. S1 of the ESI.† Relative product intensities are dependent on the metal ion; for example, MO^+^ is fairly intense for Ru^+^, whereas it is barely visible for Rh^+^. A low-intensity RhH^+^ ion was also observed, but the reaction of ground-state Rh^+^ with c-C_2_H_4_O to form RhH^+^ + CH_3_CO (the lowest energy isomer) is endothermic by 0.95 ± 0.04 eV.^40,64^ It seems possible that this species is formed by reactions with contaminants, by electronically excited Rh^+^, or by trap loading at excess kinetic energy. Overall, we conclude that these reactions are likely to mainly generate species that can be formed exothermically from ground-state reactants, but some excess excitation energy may be available as well.

### IR spectroscopy of RuCH_2_^+^

**Fig. 1:**
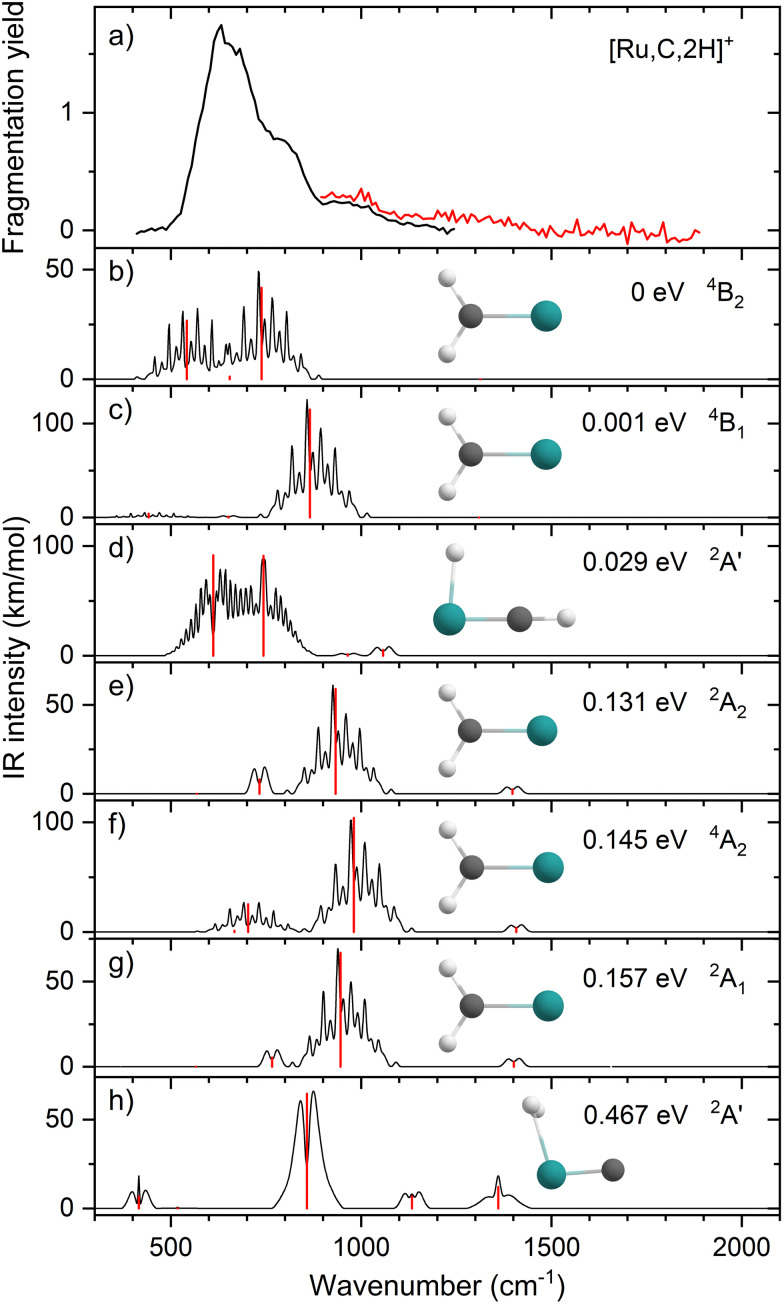
(a) Experimental IRMPD spectrum of [Ru,C,2H]^+^, the black trace is the average of four scans, the red trace is a single scan; (b)–(h) B3LYP/def2-TZVPPD calculated IR spectra of different [Ru,C,2H]^+^ states and isomers with the harmonic vibrations in red and the rovibrational envelopes in black accompanied by geometric structure, relative energy, and electronic state.

Ruthenium has a complex isotopic distribution with natural abundances at 96 (5.5%), 98 (1.9%), 99 (13%), 100 (13%), 101 (17%), 102 (32%), and 104 (19%) amu. Fig. 1(a) shows the experimental IRMPD spectrum of [Ru,C,2H]^+^. The spectrum was recorded using two FEL settings, indicated by the trace colors, with overlap between 900–1200 cm^−1^. It is constructed using only the ^101^Ru and ^102^Ru isotopes (*m*/*z* 115, 116), using RuC^+^ as the only photofragment observed (*m*/*z* 113, 114, Fig. S2a, ESI†), consistent with the dissociation thermochemistry in Table 1. Mass isolation of *m*/*z* 115, 116 prior to IR irradiation leaves small amounts of ^104^RuC^+^ (*m*/*z* 116), ^99^RuO^+^ (*m*/*z* 115), ^100^RuO^+^ (*m*/*z* 116), and ^98^RuOH_2_^+^ (*m*/*z* 116), all of which can be distinguished at the mass resolution of the FT-ICR. Because these species cannot fragment into mass channels *m*/*z* 113, 114, their presence does not affect the spectrum of [Ru,C,2H]^+^. The spectrum of [Ru,C,2H]^+^ in Fig. 1(a) shows one dominant band peaking at 637 cm^−1^. The band extends a bit further into the blue with positions of submaxima at 778 and 987 cm^−1^, identified from fitting the bands with Gaussian functions.

**Table 1:** Fragmentation channels and dissociation energies (*D*_0_) of the lowest energy [M,C,2H]^+^ species

Species	Fragments	Bond dissociation energy *D*_0_ (eV)	
		Theory^a^	Experiment
RuCH_2_^+^	RuC^+^ + H_2_	1.15	0.72 ± 0.09^b^^c^^d^
	Ru^+^ + CH_2_	3.73	3.57 ± 0.05^c^

RhCH_2_^+^	RhC^+^ + H_2_	1.29	2.76 ± 0.20^b^^e^
	Rh^+^ + CH_2_	3.60	3.69 ± 0.08,^e^ 4.08 ± 0.22^f^

a Theoretical values are calculated at the uB3LYP/def2-TZVPPD level of theory ignoring any barriers.

b Uses *D*_0_(C–H_2_) = 3.320 ± 0.001 eV.^40^

c Ref. 37.

d Ref. 65.

e Ref. 32,41.

f Ref. 42.

Fig. 1(b)–(h) shows the simulated IR spectra of different [Ru,C,2H]^+^ isomers and states calculated at the B3LYP level. Here, the ^4^B_2_ ground state (GS) is a symmetric ruthenium carbene geometry. With the convention that the *z*-axis is the symmetry axis and that the molecule lies in the *yz* plane, the ^4^B_2_ GS has a Ru–C σ bonding molecular orbital (MO) (a_1_ symmetry, using 4d_*z*^2^_ on Ru, double occupancy), a Ru–C π bond (b_1_, Ru 4d_*xz*_, double), three non-bonding MOs localized on Ru: (a_1_, 4d_*x*^2^−*y*^2^_, single; a_2_, 4d_*xy*_, double; a_1_, 5s, single), and an antibonding MO combining the Ru 4d_*yz*_ and CH_2_ orbitals (b_2_, single). Thus, the valence MO occupation (excluding the four CH_2_ bonding electrons) is (1a_1_)^2^(1b_1_)^2^(2a_1_)^1^(1a_2_)^2^(3a_1_)^1^(1b_2_)^1^. These valence molecular orbitals are shown in Fig. S3 of the ESI.†

A second electronic state, ^4^B_1_, calculated to lie only 0.001 eV higher in energy, reverses the occupation of the 4d_*x*^2^−*y*^2^_ (2a_1_) and 4d_*xy*_ (1a_2_) non-bonding orbitals, explaining the small energy difference between these two states. In another low-lying state (^4^A_2_), one electron in the non-bonding 4d_*xy*_ (1a_2_) MO is transferred to the antibonding 1b_2_ MO, leading to decreased stability, 0.15 eV above the GS. Additional carbene structures were located for different spin states. The most stable doublet-spin ruthenium carbene is ^2^A_2_, 0.13 eV above the GS with the MO occupation of (1a_1_)^2^(1b_1_)^2^(2a_1_)^2^(1a_2_)^1^(3a_1_)^0^(1b_2_)^2^. Moving an electron between the 4d_*x*^2^−*y*^2^_ and 4d_*xy*_ non-bonding orbitals results in a ^2^A_1_ state 0.16 eV above the GS. Energetics and selected orbital configurations of all stable [Ru,C,2H]^+^ species located computationally are listed in Table S1 of the ESI.†

Interestingly, early generalized valence bond (GVB) calculations of RuCH_2_^+^ by Carter and Goddard obtained a very different ordering of these states.^66^ They found a ground state of ^2^A_2_ with a nearly degenerate ^2^A_1_ state, followed by the three quartet states (^4^A_2_, ^4^B_2_, and ^4^B_1_) lying about 0.41 eV above the ground state, with a ^2^B_2_ state at 0.87 eV (see also Table S1, ESI†). However, they calculate a bond energy (*D*_e_) at their most advanced level of theory of 2.95 eV and an estimated “exact” value of 3.19 eV, which is well below the experimentally observed values. In their analysis of the bonding of these states, they characterize the bonding in the doublet spin states as covalently bound “metal–methylidenes”, whereas the quartet states are viewed as “metal–carbenes” formed by σ-donor/π-acceptor interactions.

We checked for agostic carbene geometries in the DFT calculations, but these structures converged to symmetric carbenes. More interestingly, we did find a hydrido ruthenium carbyne geometry, HRuCH^+^, with planar *C*_s_ symmetry. This species has a ^2^A′ electronic state that lies only 0.029 eV above the GS, in agreement with previous theoretical results.^37^ The ^4^A′′ hydrido carbyne was found at 1.32 eV above the GS. The lowest-energy dihydrido ruthenium carbide, HHRuC^+^, is the ^2^A′ state lying 1.72 eV above the GS. The lowest-energy dihydrogen ruthenium carbide, (H_2_)RuC^+^, is the ^2^A′ state at 0.47 eV above the GS. The lowest-energy sextet species located is a ^6^A′ state, 1.43 eV higher in energy than the GS. This species exhibits the ruthenium carbene geometry, but both hydrogen atoms are bent slightly out of plane (dihedral angle H–H–Ru–C = 8.8°).

The B3LYP-generated IR spectrum for the ^4^B_2_ state of RuCH_2_^+^ is dominated by the CH_2_ rocking and wagging modes, which are calculated at 542 and 739 cm^−1^, respectively, Fig. 1(b). These vibrations are shifted to 441 and 865 cm^−1^ for the ^4^B_1_ state, where the CH_2_ rocking-mode intensity is decreased by a factor of seven while that of the CH_2_ wagging mode is increased by a factor of three. Thus, the spectra of the ^4^B_1_ and ^4^B_2_ states of RuCH_2_^+^ differ appreciably. Interestingly, MP2 calculations enhance the intensity of the Ru–C stretch, but CCSD calculations yield an intensity similar to that obtained by B3LYP. For example, for the ^4^B_2_ GS, the frequencies in cm^−1^ (intensities, km mol^−1^) for the stretch calculated at the B3LYP/CCSD levels are 655 (1)/660 (9) compared to MP2 at 648 (53). Other RuCH_2_^+^ states have similar comparisons. It seems plausible that the experimental spectrum could be reproduced by a superposition of the five low-lying RuCH_2_^+^ states (^4^B_2_, ^4^B_1_, ^2^A_2_, ^4^A_2_, and ^2^A_1_), all of which are accessible according to the B3LYP calculations (Fig. 1) because the reaction of Ru^+^ with oxirane is exothermic by 0.20 ± 0.05 eV. At the CCSD(T)//CCSD level of theory, these states lie 0.045, 0.044, 0.150, 0.091, and 0.134 eV, respectively (Table S1, ESI†), above the ground-state HRuCH^+^ species (see next paragraph), such that all five states could be formed exothermically.

Complicating the assignment of the experimental spectrum is the low energy of the HRuCH^+^ ground state. For the HRuCH^+^ (^2^A′) species, B3LYP predicts four modes below 1500 cm^−1^: an out-of-plane C–H bending mode (743 cm^−1^, c-type), two in-plane H–M–C–H bending modes (611 and 965 cm^−1^, both with mixed a- and b-type transition character), and the M–C stretch mode (1058 cm^−1^, a-type). The M–H and C–H stretch modes are calculated to lie above 2000 cm^−1^ and are thus out of the scope of our investigation. According to B3LYP theory, only the two low-frequency modes have appreciable intensities, and these transitions fall directly in line with the observed spectrum, Fig. 1(d). For this structure, MP2 calculations again enhance the intensities of these transitions and the other two modes in the experimentally observed window. In contrast, CCSD calculations yield results in good agreement with the relative intensities predicted by B3LYP, with frequencies of 622, 751, 974, and 1036 cm^−1^, Table 2. Where the B3LYP method puts this HRuCH^+^ state 0.029 eV above the RuCH_2_^+^ (^4^B_2_) state, CCSD(T)//CCSD and EOM-SF-CCSD(fT)/EOM-SF-CCSD calculations make it the ground state, lying 0.045 eV and 0.006 eV, respectively, below the ^4^B_2_ state. The role of perturbative triples corrections was found to be critical here, as both CCSD and EOM-SF-CCSD predicted the ^4^B states to be lower in energy without these corrections. The potential energy landscape of the EOM-SF-CCSD computed states is further clarified in Fig. S4 of the ESI.†

**Table 2:** Experimental band positions and strengths (s = strong, m = medium) accompanied by theoretical calculated frequencies, intensities, and descriptions for the assigned structures

	Experiment		Theory B3LYP/CCSD/EOM^a^		Mode description
	Frequency (cm^−1^)	Strength	Frequency (cm^−1^)	Intensity (km mol^−1^)	
RuCH_2_^+^ (^4^B_2_)			542/534/*508*	27/34/*33*	CH_2_ rock
	637	s	655/660/*649*	1/9/*10*	M–C stretch
	778	s	739/731/*765*	42/49/*62*	CH_2_ wag
	987	m	1313/1343/*1336*	0/0/*2*	CH_2_ scissor

RuCH_2_^+^ (^4^B_1_)			441/516/*489*	4/22/*23*	CH_2_ rock
	637	s	651/659/*648*	1/9/*10*	M–C stretch
	778	s	865/759/*781*	115/64/*72*	CH_2_ wag
	987	m	1310/1341/*1335*	0/0/*3*	CH_2_ scissor

RuCH_2_^+^ (^2^A_2_)			568/597/*388*	0/1/*31*	CH_2_ rock
	637	s	734/739/*745*	8/0/*3*	M–C stretch
	778	s	933/924/*947*	59/65/*67*	CH_2_ wag
	987	m	1397/1413/*1360*	3/2/*0*	CH_2_ scissor

RuCH_2_^+^ (^2^A_1_)			566/593/*470*	0/1/*1*	CH_2_ rock
	637	s	766/749/*747*	8/0/*0*	M–C stretch
	778	s	946/925/*942*	59/64/*69*	CH_2_ wag
	987	m	1401/1415/*1413*	3/2/*3*	CH_2_ scissor

RuCH_2_^+^ (^4^A_2_)			703/517/*471*	25/22/*1*	CH_2_ rock
	637	s	667/647/*490*	1/8/*30*	M–C stretch
	778	s	980/766/*689*	104/58/*0*	CH_2_ wag
	987	m	1407/1352/*1334*	4/1/*13*	CH_2_ scissor

HRuCH^+^ (^2^A′)	637	s	611/622/*633*	92/97/*94*	HMCH ip bend
	778	s	743/751/*718*	91/94/*121*	C–H oop bend
	987	m	965/974/*1003*	1/1/*3*	HMCH ip bend
			1058/1036/*1102*	5/3/*3*	M–C stretch

RhCH_2_^+^ (^1^A_1_)	774	s	606/624/*600*	0/1/*1*	CH_2_ rock
	904	s	793/748/*819*	13/1/*28*	M–C stretch
	1029	s	964/971/*964*	57/65/*83*	CH_2_ wag
	1269	m	1386/1408/*1386*	4/1/*19*	CH_2_ scissor
	1783	s			Overtone

RhCH_2_^+^ (^3^A_2_)	774	s	646/627/*657*	8/21/*6*	CH_2_ rock
	904	s	674/674/*669*	21/27/*28*	M–C stretch
	1029	s	942/933/*948*	89/110/*125*	CH_2_ wag
	1269	m	1396/1431/*1425*	5/2/*2*	CH_2_ scissor
	1783	s			Overtone

a B3LYP and CCSD calculations used the def2-TZVPPD basis set. EOM-SF-CCSD calculations used the cc-pVTZ-PP basis set and ECP28MDF core potential on the metal and cc-pVTZ on the C/H atoms.

**Fig. 2:**
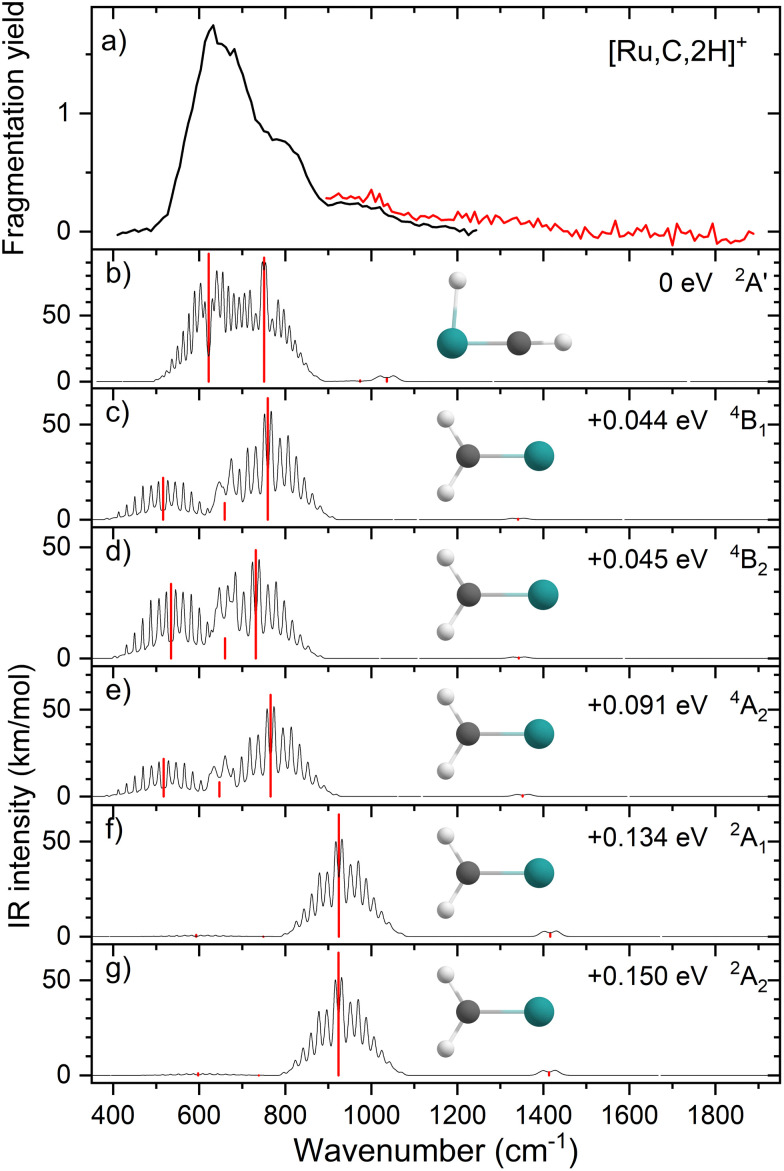
(a) Experimental IRMPD spectrum of [Ru,C,2H]^+^, the black trace is the average of four scans, the red trace is a single scan; (b)–(g) CCSD/def2-TZVPPD calculated IR spectra of different [Ru,C,2H]^+^ states and isomers with the harmonic vibrations in red and the rovibrational envelopes in black accompanied by geometric structure, relative energy, and electronic state.

**Fig. 3:**
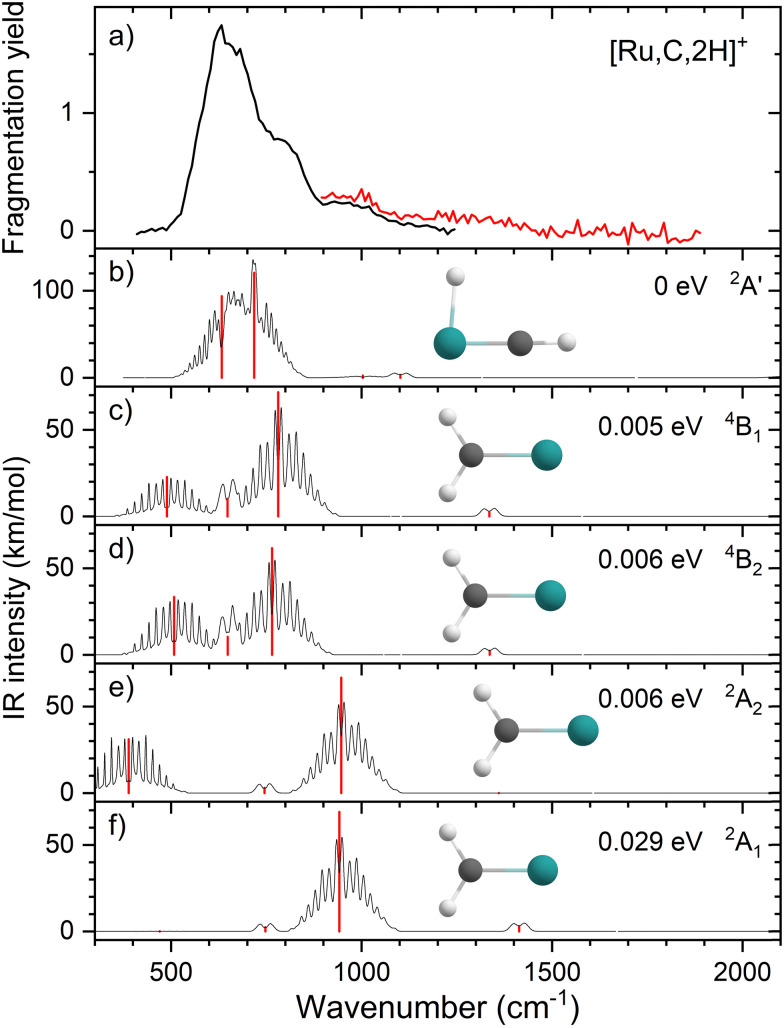
(a) Experimental IRMPD spectrum of [Ru,C,2H]^+^, the black trace is the average of four scans, the red trace is a single scan; (b)–(f) EOM-SF-CCSD/cc-pVTZ-PP calculated IR spectra of different [Ru,C,2H]^+^ states and isomers with the harmonic vibrations in red and the rovibrational envelopes in black accompanied by geometric structure, relative energy, and electronic state.

Comparisons of the predicted CCSD and EOM-SF-CCSD spectra for the low-lying states with the experimental spectrum are shown in Fig. 2 and 3, respectively. We conclude that the HRuCH^+^ species is present and captures the main feature at 637 cm^−1^, but does not capture the full shape of the 637 and 778 cm^−1^ structure. CCSD, with the pure vibrational splitting of the bright transitions in the HRuCH^+^ (^2^A′) spectrum of 622 and 751 cm^−1^, can account for both features, but does not provide the peak intensity for the 637 cm^−1^ band, whereas EOM (633 and 718 cm^−1^) is not sufficient to capture the peak at 778 cm^−1^. Moreover, neither method can explain the broad band observed at 987 cm^−1^.

We, therefore, consider additional contributors to the unique lineshape observed experimentally for RuCH_2_^+^. One option is that the ^2^A_1_ and ^2^A_2_ states account for the 987-cm^−1^ transition. For the EOM computations, the combination of the spectra for these states with that for HRuCH^+^ (^2^A′) would lead to two bands, roughly centered at 700 and 950 cm^−1^, respectively, but a large drop in the predicted spectral brightness between them (see Fig. S5 in the ESI†). For CCSD results, a more reasonable, continuous band is formed, but the intensity for the 978 cm^−1^ band is comparable to that near 637 cm^−1^. Energetically, inclusion of these A states is plausible given the 0.20 ± 0.05 eV experimental exothermicity of the Ru^+^ + oxirane reaction. A more reasonable solution is to include both ^2^A_1_/^2^A_2_ states and the ^4^B_1_/^4^B_2_ states. In both the EOM and CCSD computations, such a combination appears satisfactory to explain the observed spectral substructure, although a minor point of criticism is predicted intensity below 500 cm^−1^ (originating from the ^4^B_1_/^4^B_2_ states), in contrast to the experimental spectrum (Fig. S5, ESI†). It seems likely that an enhanced contribution of the ground state HRuCH^+^ (^2^A′) would provide an even better reproduction of the experimental bands observed and also suppress the tail to the red of 500 cm^−1^. A final, unambiguous assignment of this spectrum remains challenging because of the rotational widths and possibility of multiple-photon effects.

### IR spectroscopy of RhCH_2_^+^

**Fig. 4:**
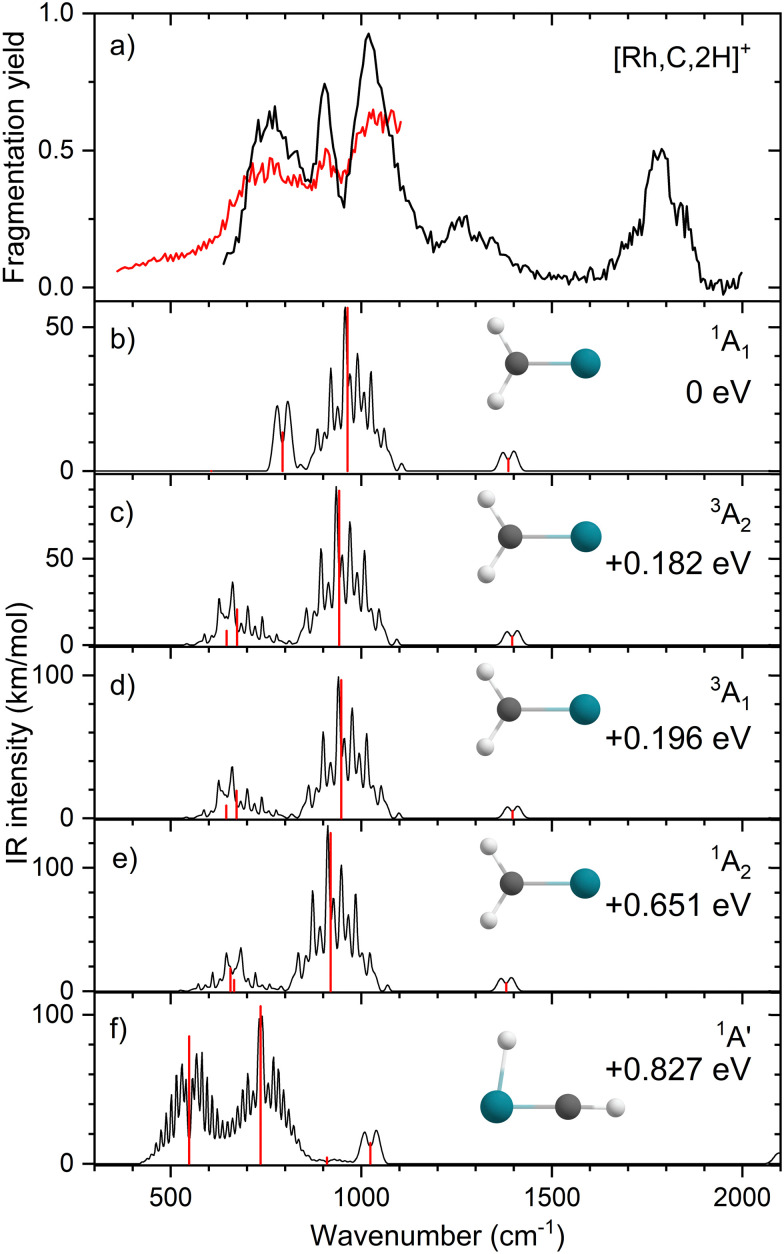
(a) Experimental IRMPD spectrum of [Rh,C,2H]^+^, the black trace is recorded in FTICR cell 4 (low fluence), the red trace in FTICR cell 1 (high fluence); (b)–(f) B3LYP/def2-TZVPPD calculated IR spectra of different [Rh,C,2H]^+^ states and isomers with the harmonic vibrations in black and the rovibrational envelopes in grey accompanied by geometric structure, relative energy, and electronic state.

Upon photofragmentation of [Rh,C,2H]^+^ (*m*/*z* 117), RhC^+^ was the only fragment observed, as shown in Fig. S2b (ES), consistent with the thermochemistry in Table 1. The experimental IRMPD spectrum of [Rh,C,2H]^+^ is shown in Fig. 4(a). The spectra were recorded in cell 4 (black trace, lowest IR fluence) and cell 1 (red trace, higher IR fluence). In contrast to the more poorly resolved, one-band-dominated spectrum for Ru, we observed five distinct IR bands: a triad of three strong bands at 774, 904, and 1029 cm^−1^, with a relatively well-resolved satellite band of medium intensity at 1269 cm^−1^ and a clear, relatively intense band at 1783 cm^−1^. The five experimental bands for the rhodium species are more than the three bands observed for the ruthenium species, which could potentially indicate the formation of multiple species. Also, the narrow width of the band observed at 904 cm^−1^ is striking as it is much sharper than the bands observed for the Ru species. The structure observed under the lower fluence conditions (black trace) slightly broadens when using a significantly increased laser fluence (red trace) but remains clearly distinguishable, which is at odds with the Ru species where only drastic broadening was observed at higher fluence. In addition, the rhodium species has experimental bands at much higher frequencies than found for [Ru,C,2H]^+^.

Fig. 4(b)–(f) shows the B3LYP-simulated IR spectra of different [Rh,C,2H]^+^ isomers and states. The ^1^A_1_ ground state has the symmetric rhodium carbene geometry with *C*_2v_ symmetry and a valence MO occupation of (1a_1_)^2^(1b_1_)^2^(2a_1_)^2^(1a_2_)^2^(3a_1_)^0^(1b_2_)^2^. This configuration is markedly different from the Ru case, as this species has a ground state with multiple unpaired electrons on the metal center, including an electron in the 3a_1_ (5s-like) orbital. In contrast, the RhCH_2_^+^ GS clearly has no unpaired electrons nor any electrons in the 5s orbital. Identification of the true ground state was complicated by finding another ^1^A_1_ state that differed from that shown because the 2a_1_ (4d_*x*^2^−*y*^2^_) and 1a_2_ (4d_*xy*_) orbitals were mixed, leading to a lower energy by 0.020 eV. This outcome is an artifact of the B3LYP calculation, as demonstrated by the EOM calculations described below.

The lowest-energy triplet species for [Rh,C,2H]^+^ according to the B3LYP method is a symmetric carbene with a ^3^A_2_ state and lies only 0.18 eV above the GS. This ^3^A_2_ state has one electron in the 4d_*xy*_ (1a_2_) orbital, two electrons in the 4d_*x*^2^−*y*^2^_ (2a_1_) orbital, and one electron in the 5s (3a_1_) orbital. A ^3^A_1_ state, slightly higher in energy at 0.20 eV above the GS, has two electrons in the 4d_*xy*_ orbital, one electron in the 4d_*x*^2^−*y*^2^_ orbital, and one electron in the 5s orbital. We find two singlet carbene states with 5s occupancy at 0.65 eV (^1^A_2_) and 0.66 eV (^1^A_1_), Table S1 (ESI†), and the lowest-energy quintet at 1.78 eV above the GS. Hydrido rhodium carbyne structures are found at 0.83 eV (^1^A′) and 1.53 eV (^3^A′) above the GS. The lowest-energy dihydrido rhodium carbide (^3^A′′) lies 3.42 eV above the GS, while the lowest energy dihydrogen rhodium carbide (^1^A′) is 0.79 eV above the GS. The energetically preferred geometry is thus clearly a symmetric carbene.

The calculated IR spectra of the ^1^A_1_ and ^3^A_2_ states exhibit moderately intense bands for the Rh–C stretch (a narrow a-type transition, 13 and 21 km mol^−1^) and strong bands for the out-of-plane CH_2_ wag (a broad b-type transition, 57 and 89 km mol^−1^), whereas the in-plane CH_2_ rock and scissors modes are relatively weak with intensities <10 km mol^−1^. The two states differ mainly in the frequency of the Rh–C stretch vibration, which is 793 cm^−1^ for the ^1^A_1_ GS and 674 cm^−1^ for the ^3^A_2_ state, Table 2. Both states have similar CH_2_ wag frequencies of 964 and 942 cm^−1^, respectively. In comparison to the Ru carbene cation, the Rh–C stretch vibration is calculated to have a significantly higher IR intensity. The three intense bands calculated at 674, 793, and 964/942 cm^−1^ could conceivably match the three main peaks observed experimentally, but they are systematically shifted to lower frequencies. The band observed at 1269 cm^−1^ could be associated with the RhCH_2_^+^ scissor vibration, but conceivably has components of overtones of the in-plane CH_2_ rock (606/646 cm^−1^). Likewise, the band observed at 1783 cm^−1^ seems likely to be either a combination band or an overtone, as found earlier for a similar system.^19^ An anharmonic frequency calculation, using the second-order vibrational perturbation theory (VPT2) method^67,68^ and the same B3LYP/def2-TZVPPD potential energy surface, places the CH_2_ wagging overtone at 1892 cm^−1^ with an intensity of 1.3 km mol^−1^, and the combination band of the CH_2_ rocking and scissoring modes at 1918 cm^−1^ with a similar intensity. The strong intensity with which this band is experimentally found is remarkable, but it could be attributed to a higher IRMPD efficiency for higher photon energies.

Fig. 4(f) shows the spectrum calculated for the hydrido carbyne HRhCH^+^ species, which has a ^1^A′ ground state. This species is found at fairly high energy, 0.83 eV above RhCH_2_^+^ (^1^A_1_), which would make its formation unlikely. Further, it exhibits two strong bands at 548 and 736 cm^−1^ that do not match the experimental spectrum. From a spectroscopic point of view, the possibility that this species has a small population cannot be eliminated, in particular because the predicted low-frequency band could explain the small intensity near 500 cm^−1^ observed in the high-intensity (red) IR spectrum. However, it is clear that the majority of the spectrum must be associated with low-energy carbene species. This conclusion is even more evident from the more advanced theoretical results found in the next section.

### EOM-SF-CCSD results for RhCH_2_^+^

**Fig. 5:**
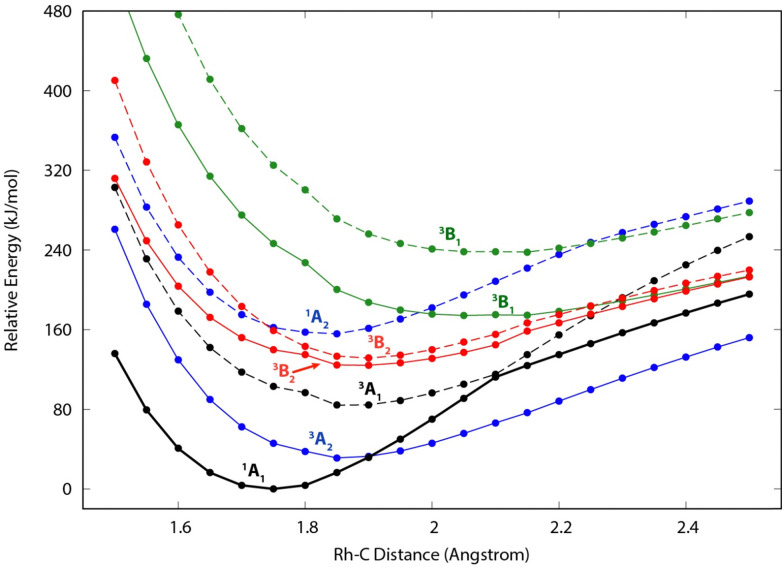
Constrained potential energy curves along the metal–carbon distance in RhCH_2_^+^ in *C*_2v_ symmetry, as calculated at the EOM-SF-CCSD/cc-pVTZ-PP level of theory. Solid (dashed) lines depict the lowest-energy (first excited) state of each symmetry. The structure was optimized in the lowest state of *A*_1_ symmetry, and all other state energies represent vertical excitations at the same structure. Spin and symmetry labels for each state, corresponding to the assignments at the ^1^A_1_ minimum geometry, are provided for each curve. [The symmetry labels for the B_1_ and B_2_ states are reversed from the Q-Chem outputs, in order to align with the symmetry-axis conventions of Gaussian computations in the remainder of this work.]

Because of the unusual spectrum for the [Rh,C,2H]^+^ species and the difficulty in assigning the ground state computed with the B3LYP level of theory, we also explored more advanced EOM-SF-CCSD calculations. The potential energy curves along the Rh–C coordinate, shown in Fig. 5, indicate a manifold of electronic states for the carbene species with many curve crossings (with relative energies at the equilibrium Rh–C distances listed in Table 3). In particular, the ^1^A_1_ ground-state potential crosses the ^3^A_2_ potential near 1.9 Å, which also nearly coincides with the minimum in the latter state. Furthermore, the ground state also exhibits an avoided crossing with the next-lowest A_1_ state (^3^A_1_) near 2.1 Å. Such interactions are likely to explain why the single-reference calculations encountered substantial difficulties in establishing the structures and electronic state of this species. Even the ground ^1^A_1_ state exhibited notable open-shell singlet character in these EOM-SF computations, with Mulliken spin populations (using the EOM-SF-CCSD wavefunctions) of −0.38 and +0.38 on the Rh and CH_2_ subunits, respectively. This outcome is consistent with the spin density depicted in Fig. S6 of the ESI.† Notably, although previous computational studies of RhCH_2_^+^ correctly identified the ^1^A_1_ ground state and ^3^A_2_ first excited state,^43,44^ the role of multireference character in the ground state has not been fully appreciated, particularly as the Rh^+^–CH_2_ bond is stretched.

**Table 3:** Energies of [Ru,C,2H]^+^ and [Rh,C,2H]^+^ species calculated at the EOM-SF-CCSD/cc-pVTZ-PP level of theory. EOM-SF-CCSD(fT) energies are provided in square brackets

Structure	State	*E*_rel_ (eV)	*E*_rel_ + ZPE (eV)	〈*S*^2^〉
RuCH_2_^+^	^4^B_1_	0.000	0.000 [0.005]	4.098
	^4^B_2_	0.001	0.001 [0.006]	4.101
	^2^A_2_	0.010	0.012 [0.006]	0.967
	^2^A_1_	0.021	0.036 [0.029]	0.938
	^4^A_2_	1.036	1.018 [1.013]	2.835^a^
HRuCH^+^	^2^A′	0.119	0.072 [0.000]	0.967

RhCH_2_^+^	^1^A_1_	0.000	0.000 [0.000]	0.244
RhCH_2_^+^	^3^A_2_	0.296	0.295 [0.312]	2.251
(H_2_)RhC^+^ (*C*_s_)	^1^A_1_	0.795	0.636 [0.570]	0.149
RhCH_2_^+^	^3^A_1_	0.833	0.833 [0.764]	1.556^a^
HRhCH^+^	^1^A′	0.947	0.895 [0.785]	0.256
RhCH_2_^+^	^3^B_2_	1.271	1.242 [1.185]	1.355^a^
RhCH_2_^+^	^3^B_2_	1.358	1.355 [1.272]	1.137^b^
RhCH_2_^+^	^1^A_2_	1.594	1.598 [1.569]	0.277
RhCH_2_^+^	^3^B_1_	1.761	—c	1.546^a^
HRhCH^+^	^3^A′′	2.080	2.012 [1.958]	2.011
RhCH_2_^+^	^3^B_2_	2.430	2.392 [2.315]	1.493^a^
(H_2_)RhC^+^ (*C*_s_)	^3^A′′	3.189	3.020 [2.878]	1.144^a^

a Significant remnant spin contamination in EOM-SF-CCSD state.

b Species corresponds to a transition state in *C*_2v_ symmetry.

c Finite-difference frequency alternated electronic states; no ZPE correction available.

**Fig. 6:**
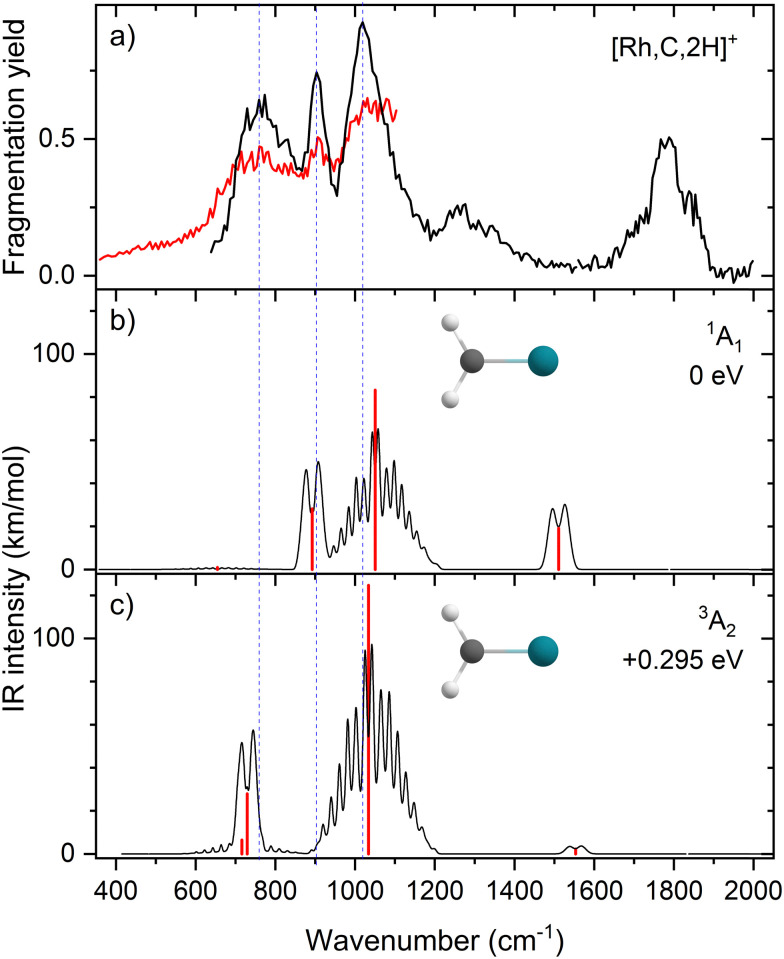
(a) Experimental IRMPD spectrum of [Rh,C,2H]^+^; (b) harmonic stick spectrum (in red) of the RhCH_2_^+^ (^1^A_1_) structure at the EOM-SF-CCSD level and rovibrational simulations (in black) with frequencies scaled by 1.09; (c) same as (b) for RhCH_2_^+^ (^3^A_2_).

The EOM results identified eleven distinct species (some of which are excited states of a given symmetry): several RhCH_2_^+^ carbenes along with (H_2_)RhC^+^ (^1^A_1_) and HRhCH^+^ (^1^A′ and ^1,3^A′′) structures (Table 3). On the basis of these calculations, the ^1^A_1_ and ^3^A_2_ carbenes seem to be the likely candidates for formation in the experiments conducted here. The vibrational spectra of these two states are similar to those found at the B3LYP level, although the Rh–C stretch intensity is notably enhanced, particularly in the ground-state singlet, as listed in Table 2. The combination of these two species results in a spectrum that reproduces the three strong bands observed experimentally, as shown in Fig. 6 where the frequencies have been scaled by a factor 1.09, resulting in a blue-shift of about 90 cm^−1^. (The scaling factor of 1.09 is chosen to best match the experimental spectrum. The comparison to the unshifted spectrum is shown in Fig. S7 of the ESI.†) The spectrum computed for the ^3^A_2_ carbene may even rationalize the larger spectral width of the band observed at 774 cm^−1^ compared to the 904 cm^−1^ band. This is because the Rh–C stretch for the ^1^A_1_ species is isolated whereas the same mode for the ^3^A_2_ state, at 668 cm^−1^, nearly overlaps with the more intense CH_2_ rocking mode. Cooperative excitation of these bands could lead to the broadening observed, especially in the high-fluence spectrum. The assignment of the additional bands observed experimentally at higher wavenumbers remains inconclusive but seems likely to be the overtones identified above, similar to the overtone band previously found in the spectrum of PtCH_2_^+^.^18,19^

Although a systematic methodology comparison is not the focus of the present study, we note anecdotally that the more recently developed, range-separated hybrid functional ωB97M-V^69^ was found to exhibit harmonic frequencies and intensities that are notably more closely aligned with the EOM-based spectra (Fig. S8 of the ESI†). This functional may serve as a more accurate, cost-efficient method for future studies of this complex, although the state selectivity of the EOM methods provides considerably more control over the targeted electronic states.

Given the relative success of this alternative functional for [Rh,C,2H]^+^, the spectra of [Ru,C,2H]^+^ were also reexamined with ωB97M-V/cc-pVTZ-PP. The density functional encouragingly exhibited harmonic spectra for [Ru,C,2H]^+^ (Fig. S9 of the ESI†) that were quite similar to those computed by CCSD (Fig. 2). However, the energy of the HRuCH^+ 2^A′ state was computed to be more than 0.6 eV above the ground ^4^B_2_ state, in stark contrast to the results of CCSD(T) and EOM-SF-CCSD(fT) where it is the ground state structure. Simultaneously capturing both the overall energy landscape and the nuanced shape of the potential energy surface near equilibrium evidently remains a daunting challenge for density functional theory for these ionic transition-metal complexes.

### DFT performance for prediction of M–C stretch vibrational intensities

Judging from the comparison between the B3LYP-, CCSD-, and EOM-SF-CCSD-calculated spectra for RuCH_2_^+^ (Table 2), the drastic underestimation of the IR intensities identified for the FeCH_2_^+^ and CoCH_2_^+^ species^45^ remains evident for this system. For the quartet spin carbenes, B3LYP predicts intensities for the Ru–CH_2_ stretch vibration that are an order of magnitude smaller than the CCSD and EOM-SF-CCSD calculations. Only for the ^2^A_2_ state does B3LYP predict an intensity for this stretch that is comparable to the EOM-SF-CCSD result. However, the concluded presence of the HRuCH^+^ species makes it complex to test this observation on the basis of comparison with experiment. Because the latter species is identified from dominant bands associated with bending modes, which are less sensitive to potential underestimations of charge separation, the RuCH_2_^+^ species cannot form a good theoretical benchmark.

On the other hand, comparison of the computed spectra for the ^1^A_1_ and ^3^A_2_ RhCH_2_^+^ species suggests that the single-reference B3LYP method does not underperform compared to the CCSD/EOM approaches for frequencies (Table 2). Comparison with experiment would require an anomalous scaling factor for the B3LYP spectra compared with previous work. For IR intensities, the enhanced intensities of the Rh–C stretch found by EOM-SF-CCSD method helped solidify the assignment of the spectra (in particular, the bands at 774 and 904 cm^−1^). Of course, the vicinity of so many low-lying electronic states clearly complicates finding the experimental ground states.

### Group 8 and 9 trends: carbene *versus* hydrido carbyne structures

In previous work, we have presented the experimental IRMPD spectra of [Os,C,2H]^+^ and [Ir,C,2H]^+^.^18–20,22^ We concluded that the osmium species had the hydrido carbyne HOsCH^+^ structure, as did the iridium species, which exhibited small amounts of a higher-energy, symmetric carbene, as well. If we put the current assignments for Ru and Rh in perspective with these previous findings, we note that both 5d elements prefer the formation of a hydrido carbyne structure, whereas the 4d Ru forms both this and a carbene species and Rh yields only the carbene. As shown in Table 4, the 5d hydrido carbynes are energetically preferred, but more strongly so for the group 8 Os case. This observation is consistent with the assignment of HOsCH^+^ (^2^A′), whereas part of the product for iridium (group 9) is assigned to a carbene structure (0.3 or 0.7 eV higher in energy). This trend appears to hold for 4d elements, where the RuCH_2_^+^ (^4^B_2_) carbene is competitive with the ^2^A′ HRuCH^+^ hydrido carbyne, whereas HRhCH^+^ (^1^A′) is significantly higher in energy than RhCH_2_^+^ (^1^A_1_). Intrinsically, the 5d TMs have stronger bonds to C and H than the 4d TMs, *e.g.*, for group 9, see Fig. 4 in ref. 13. (For group 8, see ref. 16, 37, 70 and 65). Because HMCH^+^ has four covalent bonds to the metal whereas MCH_2_^+^ only has two covalent M–C bonds, the more highly coordinated hydrido carbyne structure becomes more favorable for 5d relative to 4d TMs.

**Table 4:** Theoretical energies (eV) of HMCH^+^ species relative to the most stable MCH_2_^+^ carbene

M	B3LYP	CCSD(T)//CCSD
Ru	0.03	−0.005
Rh	0.80	0.76
Os	−0.50	−0.82
Ir	−0.30	−0.69

**Fig. 7:**
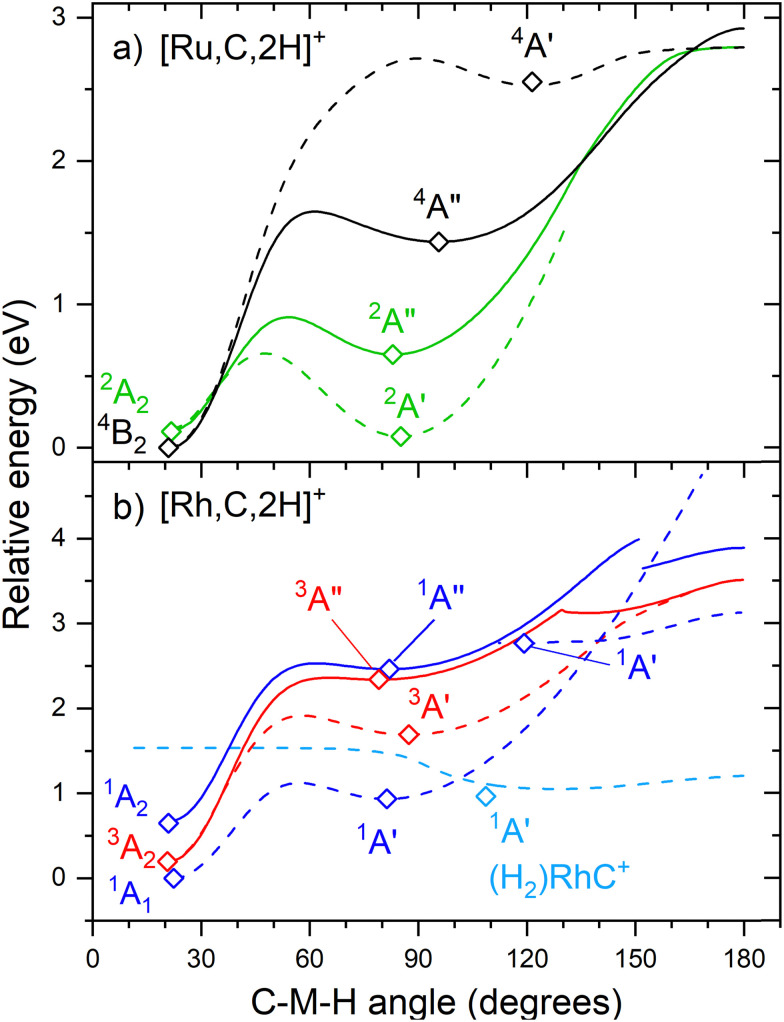
Potential energy surfaces of [M,C,2H]^+^ along the C–M–H angle calculated at the uB3LYP/def2-TZVPPD level of theory with steps of 1°. Open diamonds indicate calculated structures that successfully converged to true minima at the indicated C–M–H angle. Solid lines indicate A′′ states while A′ states are indicated by dashed lines. Doublet spin surfaces are depicted in green, quartet in black, singlet in blue, and triplet in red. All energy values are relative to the GS symmetric carbene structures.

The hydrido carbyne structures for 4d and 5d elements are found on the same or a lower spin surface as that of the lowest-energy carbene structure. To investigate whether we might have missed hydrido carbyne structures, the potential energy surfaces (PESs) connecting the MCH_2_^+^ and HMCH^+^ structures were calculated for M = Ru and Rh (Fig. 7) with B3LYP. PESs were generated by starting from a symmetric carbene structure and systematically increasing the C–M–H angle while the rest of the molecule was allowed to relax. The PES scans were generated for both the A′ and A′′ configurations in dashed and solid lines, respectively. The PESs for Ru and Rh show clear minima and appreciable barriers for H migration from the metal to carbon. Here again, the higher stability of carbyne structures for Ru in comparison to Rh is clearly visible.

## Conclusion

Reacting the 4d group 8 and 9 transition metal ions M^+^ (M = Ru and Rh) with oxirane in a room temperature ion trap led to the formation of [M,C,2H]^+^. These species were mass-isolated in the FTICR mass spectrometer coupled to the infrared intracavity free-electron laser FELICE, where they were spectroscopically characterized. The IRMPD spectrum of [Ru,C,2H]^+^ contains one broad band around 700 cm^−1^, whereas the IRMPD spectrum of [Rh,C,2H]^+^ is characterized by much sharper, distinct bands. On the basis of B3LYP and CCSD(T)//CCSD energetics, the [Rh,C,2H]^+^ species is expected to be a carbene, and for [Ru,C,2H]^+^, a competitive hydrido carbyne structure is also found. Comparison of the experimental IRMPD spectra with the predictions from B3LYP calculations offers moderate agreement. Calculations at the CCSD and EOM-SF-CCSD levels offer a modest improvement, but also show how sensitive an experimental constraint the spectroscopic fingerprint provides for these electronically complex systems. These comparisons suggest the presence of the HRuCH^+^ species, with contributions from RuCH_2_^+^ carbenes. The case of [Rh,C,2H]^+^ offers a similar complexity. Despite the much more structured experimental IR spectrum, assignment based on either B3LYP or conventional CCSD simulations is difficult. Because population of the energetically close singlet and triplet spin surfaces could significantly influence the behavior of this species and because of moderately strong state mixing within the singlet manifold, EOM-SF-CCSD calculations were performed. These computational results suggest that a combination of the (partially open-shell) ^1^A_1_ and ^3^A_2_ carbenes can explain the complex structure of the [Rh,C,2H]^+^ spectrum. Notably, including the multireference character of this species was critical in properly assigning the spectrum, but a modest shift in the calculated harmonic frequencies was still required to match experiment.

## Conflicts of interest

There are no conflicts of interest to declare.

## Supplementary Material

CP-026-D4CP00012A-s001
