# Supplementary material for: IR spectroscopic characterization of [M,C,2H]+ (M = Ru and Rh) products formed by reacting 4d transition metal cations with oxirane: Spectroscopic evidence for multireference character in RhCH2+
Source: Phys Chem Chem Phys. 2024 Mar 15;26(15):11445–58. doi: 10.1039/d4cp00012a (PMC11022548; doi:10.1039/d4cp00012a)
Supplement: CP-026-D4CP00012A-s001 [file CP-026-D4CP00012A-s001.pdf]

Electronic Supplementary Material (ESI) for Physical Chemistry Chemical Physics.
This journal is © the Owner Societies 2024

IR spectroscopic characterization of $[M,C,2H]^+$ (M = Ru and Rh) products formed by reacting 4d transition metal cations with oxirane: Spectroscopic evidence for multireference character in $RhCH_2^+$

Frank J. Wensink,[1] Corry E. Smink,[1] Brandon C. Stevenson,[2] Ryan P. Steele,[2] Joost M. Bakker,[1] and P. B. Armentrout[2]

[1]Radboud University, Institute for Molecules and Materials, FELIX Laboratory, Toernooiveld 7, 6525 ED Nijmegen, The Netherlands
[2]University of Utah, Department of Chemistry, 315 South 1400 East, Salt Lake City, Utah 84112, United States

Supporting Information

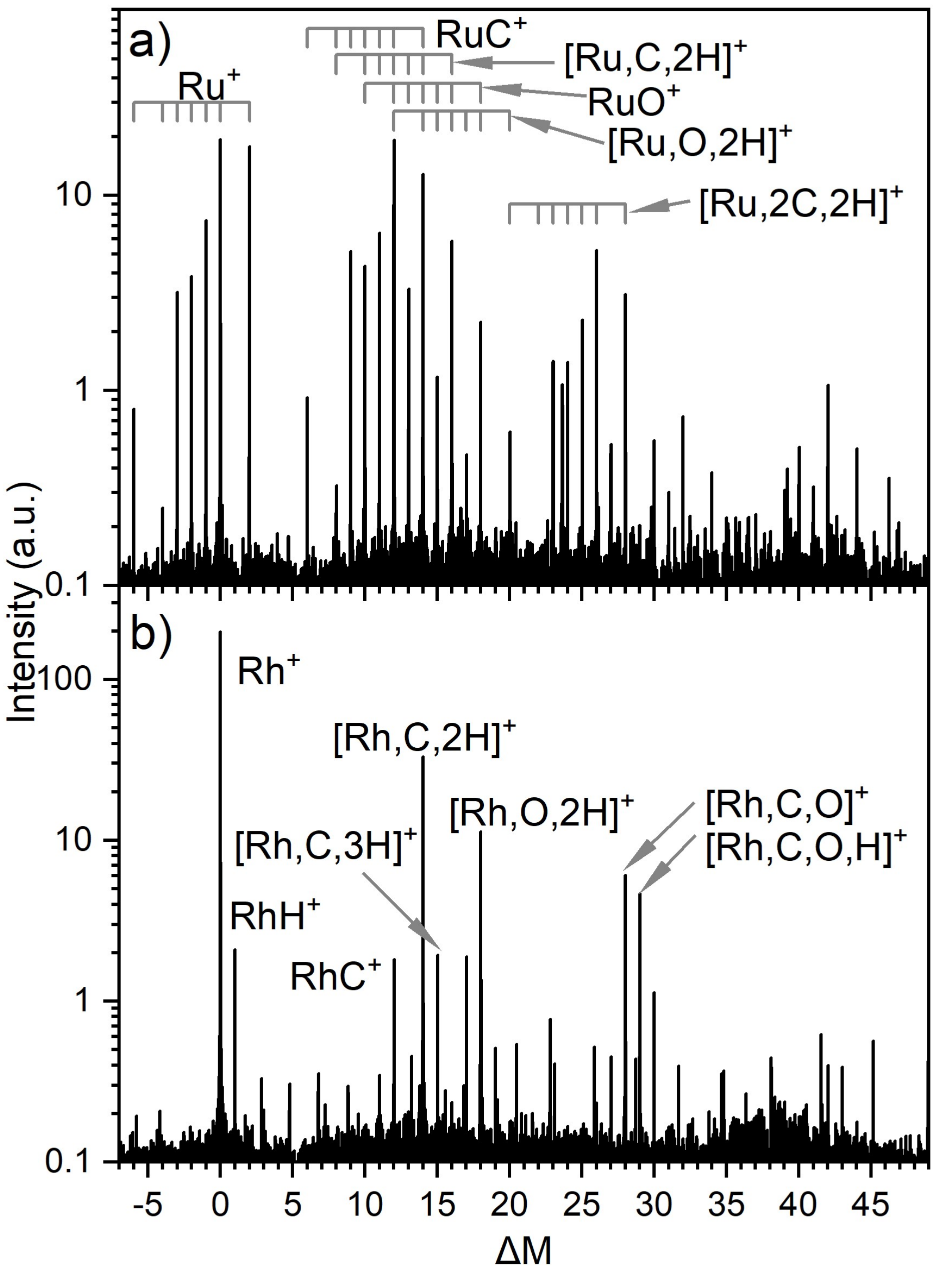

Figure S1: Product mass distributions of the reaction between ethylene oxide and a) Ru^+ (ΔM = *m/z* – 101.907) and b) Rh^+ (ΔM = *m/z* – 102.907) in the room temperature ion trap.

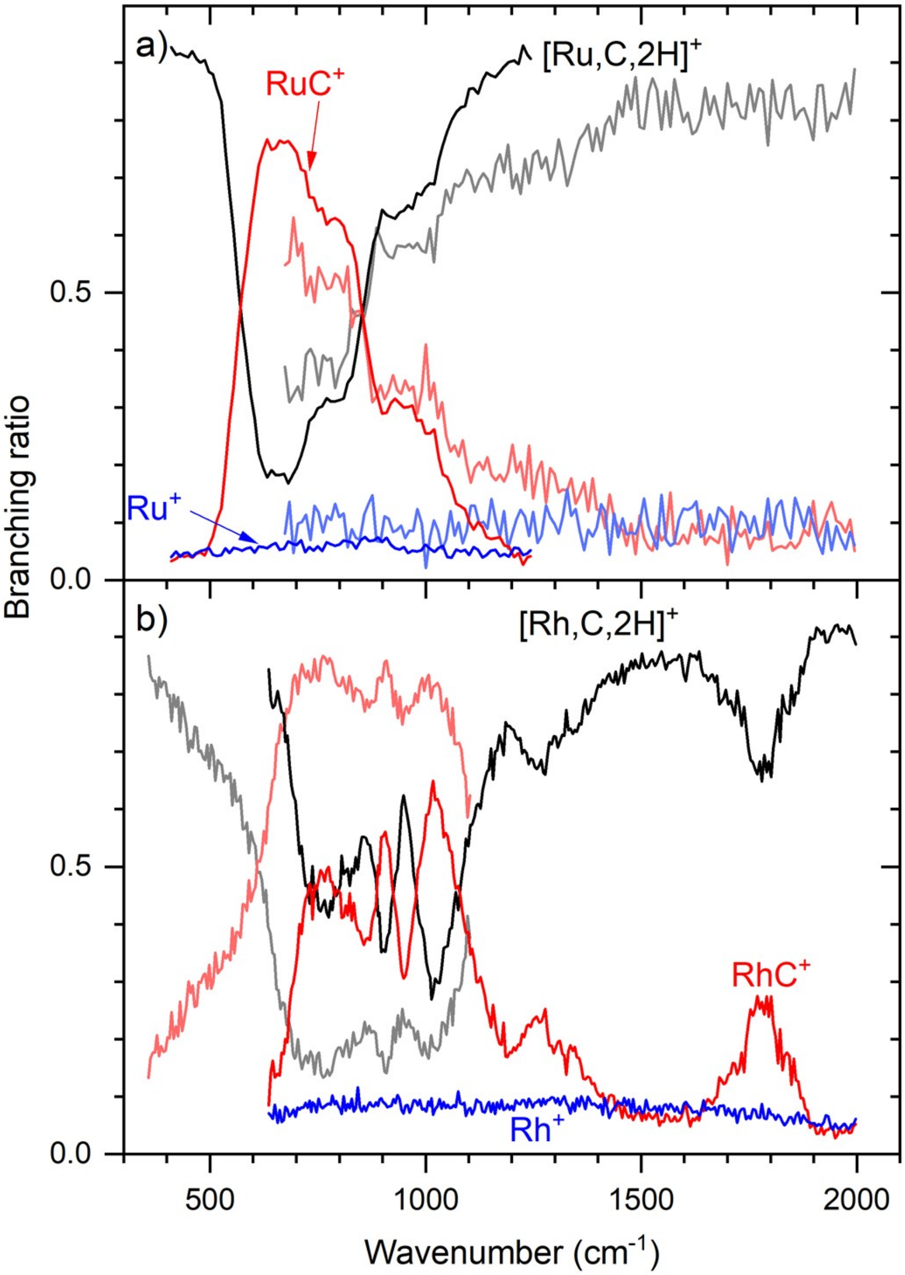

Figure S2: Branching ratios of $[M,C,2H]^+$ (in black) and its photofragmentation products, MC^+ (in red) and bare M^+ (in blue), for M = Ru (panel a) and Rh (panel b). Bright versus light colors indicate measurements with different FEL settings.

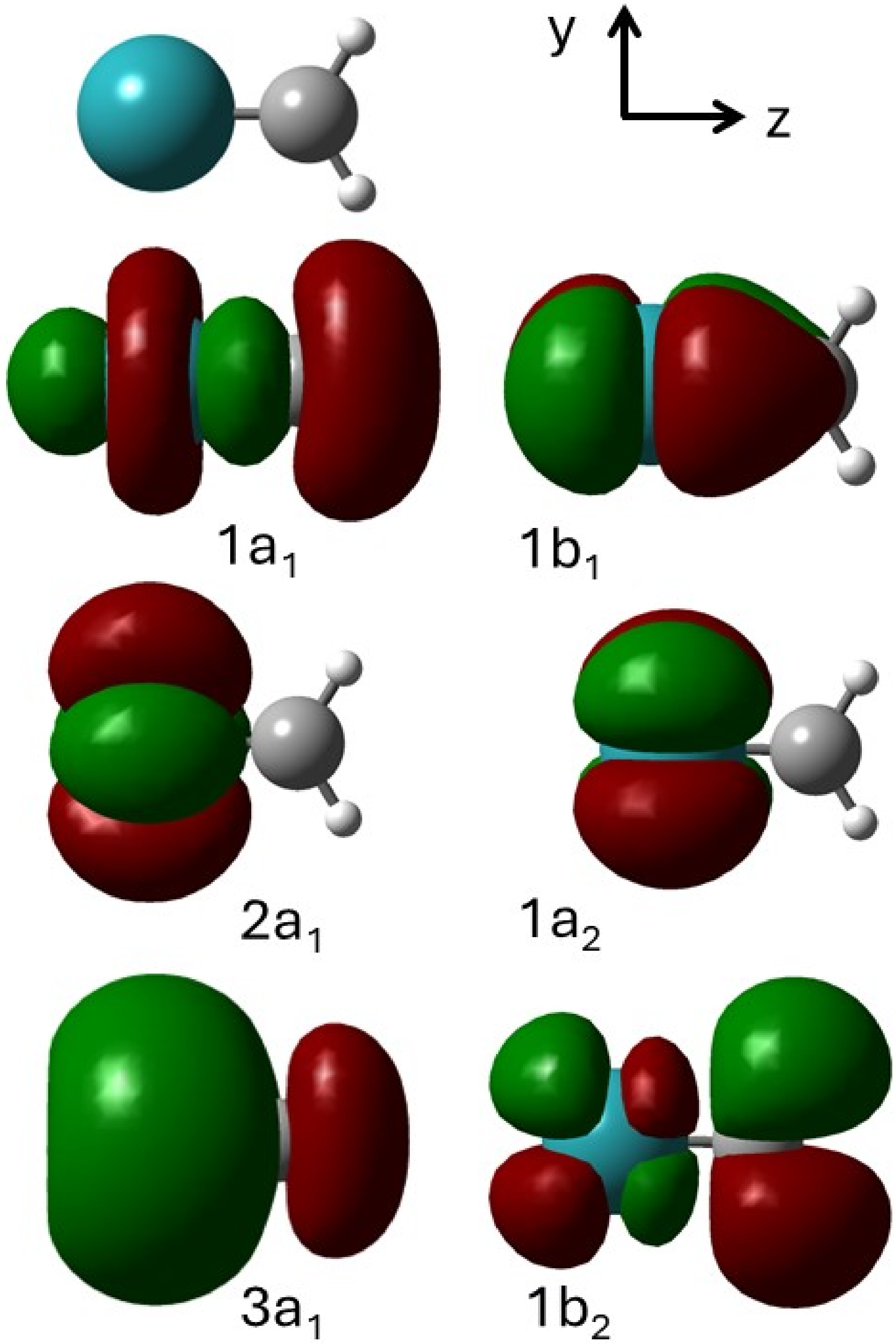

Figure S3: Valence molecular orbitals calculated for $RuCH_2^+$ ($^4B_2$) at the B3LYP/def2-TZVPPD level.

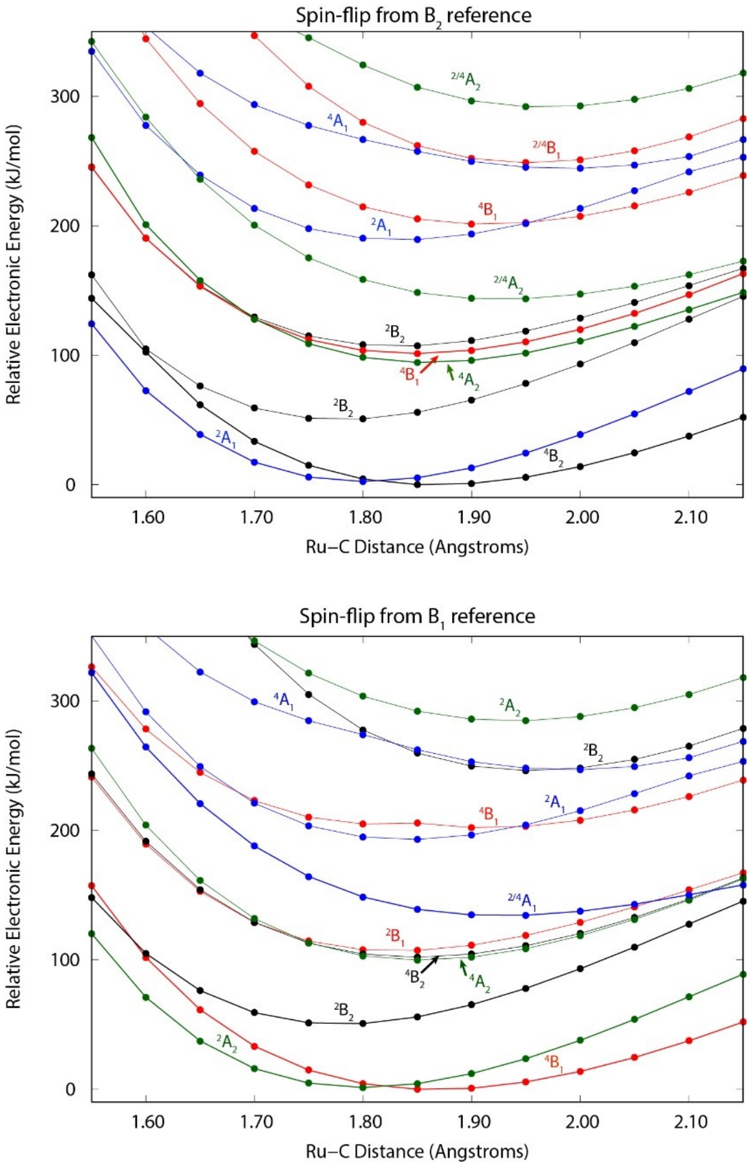

Figure S4: Constrained potential energy curves along the metal-carbon distance in $RuCH_2^+$ in C_{2v} symmetry, as calculated at the EOM-SF-CCSD/cc-pVTZ-PP level of theory. Because of the effectively doubly degenerate ground state with the HF method, spin-flip computations were performed, independently, using both the B_1 (upper panel) and B_2 (lower panel) quartet reference states. The structure was optimized in the lowest state of A_1 spin-flip transition symmetry (yielding the lowest-energy state in both symmetry cases), and all other state energies represent vertical excitations at the same structure. Spin and symmetry labels for each state, corresponding to the assignments at the $^4B_1$/$^4B_2$ minima, are provided for each curve, and curves are color-coded by state symmetry. States labelled with a “2/4” multiplicity correspond to states with sufficient spin contamination in the EOM-SF state that the multiplicity cannot be properly resolved. [Note: The B_1/B_2 state labels have been reversed from the Q-Chem outputs to align with the symmetry-axis convention of Gaussian outputs.]

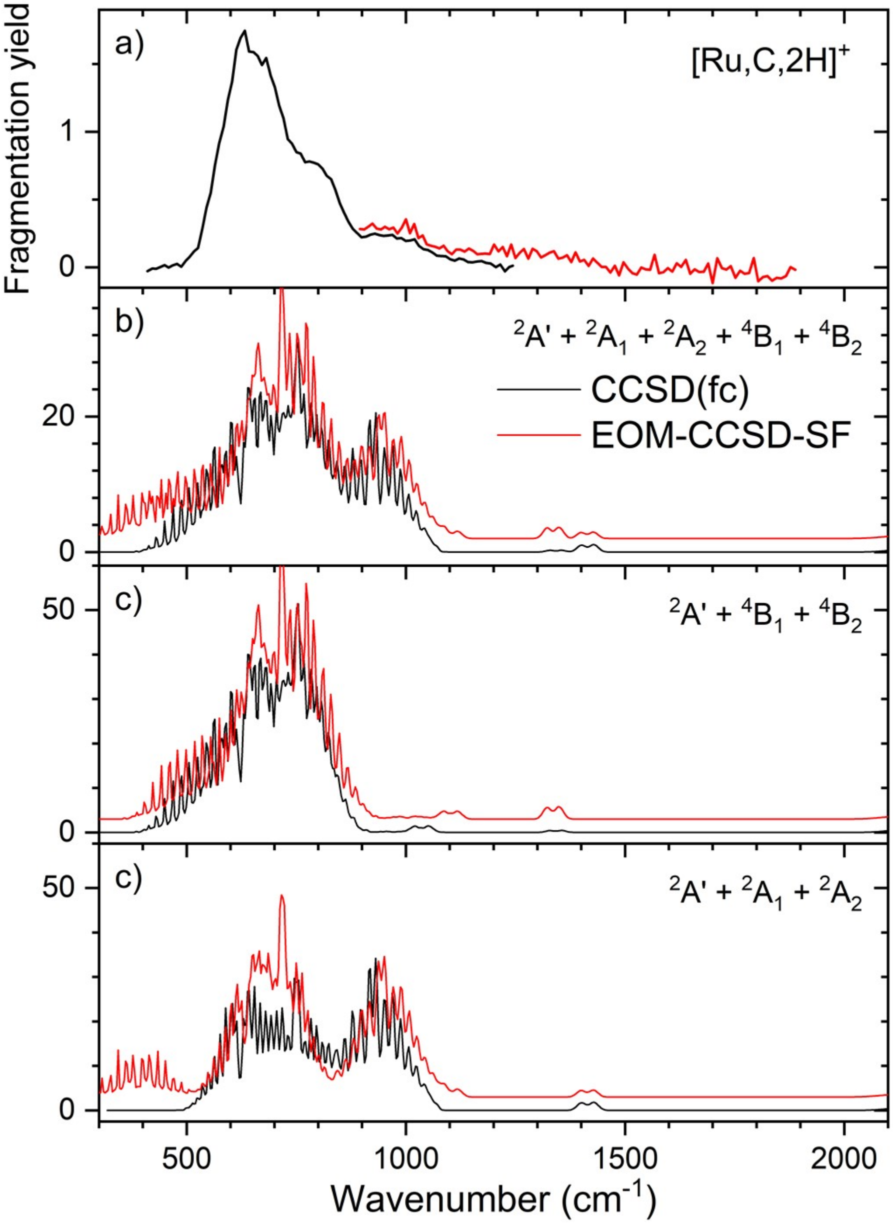

Figure S5: (a) Experimental IRMPD spectrum of $[Ru,C,2H]^+$; the black trace is the average of four scans, the red trace is a single scan. (b)-(d) Summed unscaled, harmonic IR spectra, computed with the CCSD/def2-TZVPPD (black traces) and EOM-SF-CCSD/cc-pVTZ-PP (red traces) methods for several combinations of low-lying electronic states of $RuCH_2^+$ and $HRuCH^+$. Panel b): $^2A'$, $^2A_1$, $^2A_2$, $^4B_1$, and $^4B_2$; panel c): $^2A'$, $^4B_1$, and $^4B_2$; panel d: $^2A'$, $^2A_1$, and $^2A_2$. Individual spectra are not weighted before summation.

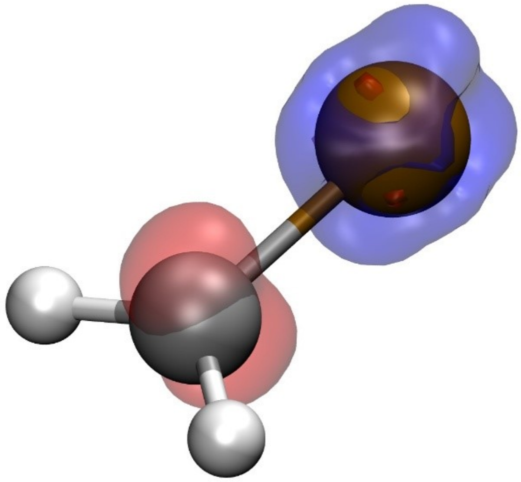

Figure S6: Spin density in the ground electronic state ($^1A_1$) of $RhCH_2^+$, as computed with the EOM-SF-CCSD/cc-pVTZ-PP method.

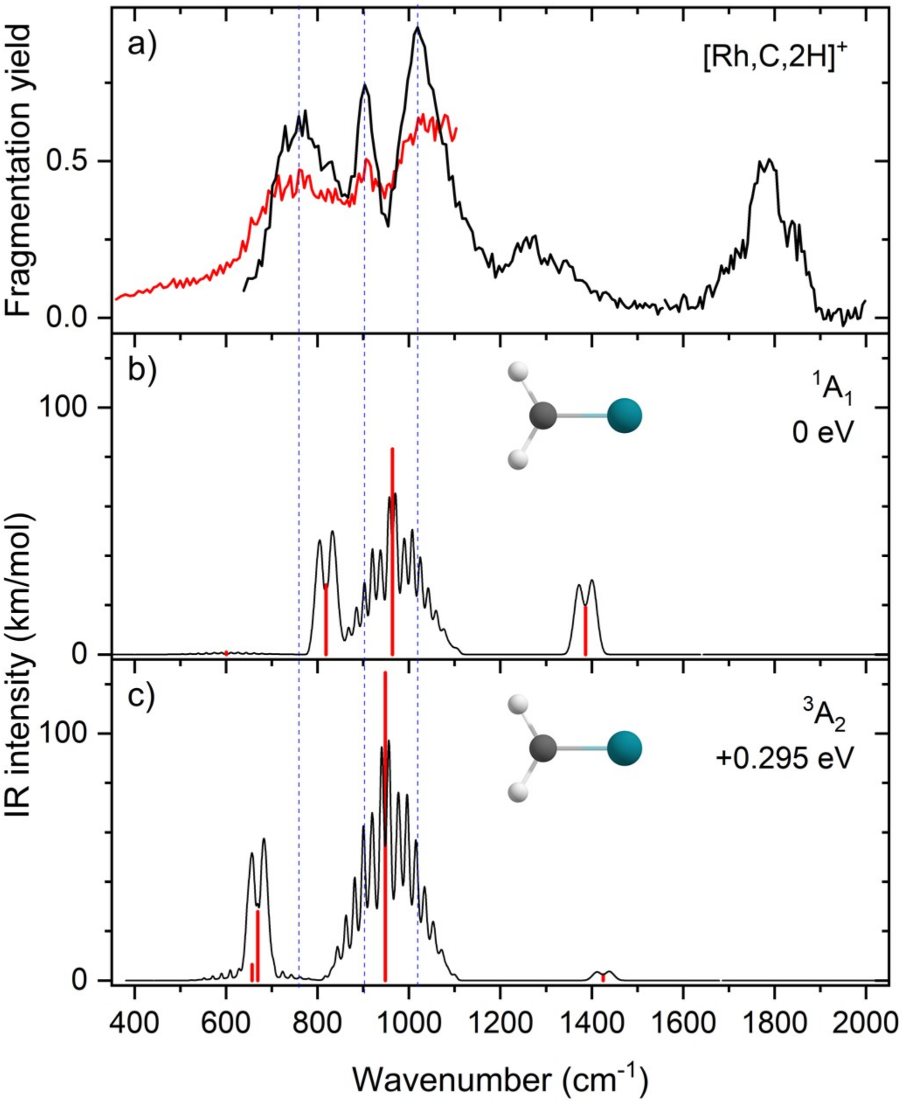

Figure S7: (a) Experimental IRMPD spectrum of $[Rh,C,2H]^+$; b) Harmonic stick spectrum of the $RhCH_2^+$ ($^1A_1$) structure at the EOM-SF-CCSD level and rovibrational simulations; c) same as b) for $RhCH_2^+$ ($^3A_2$).

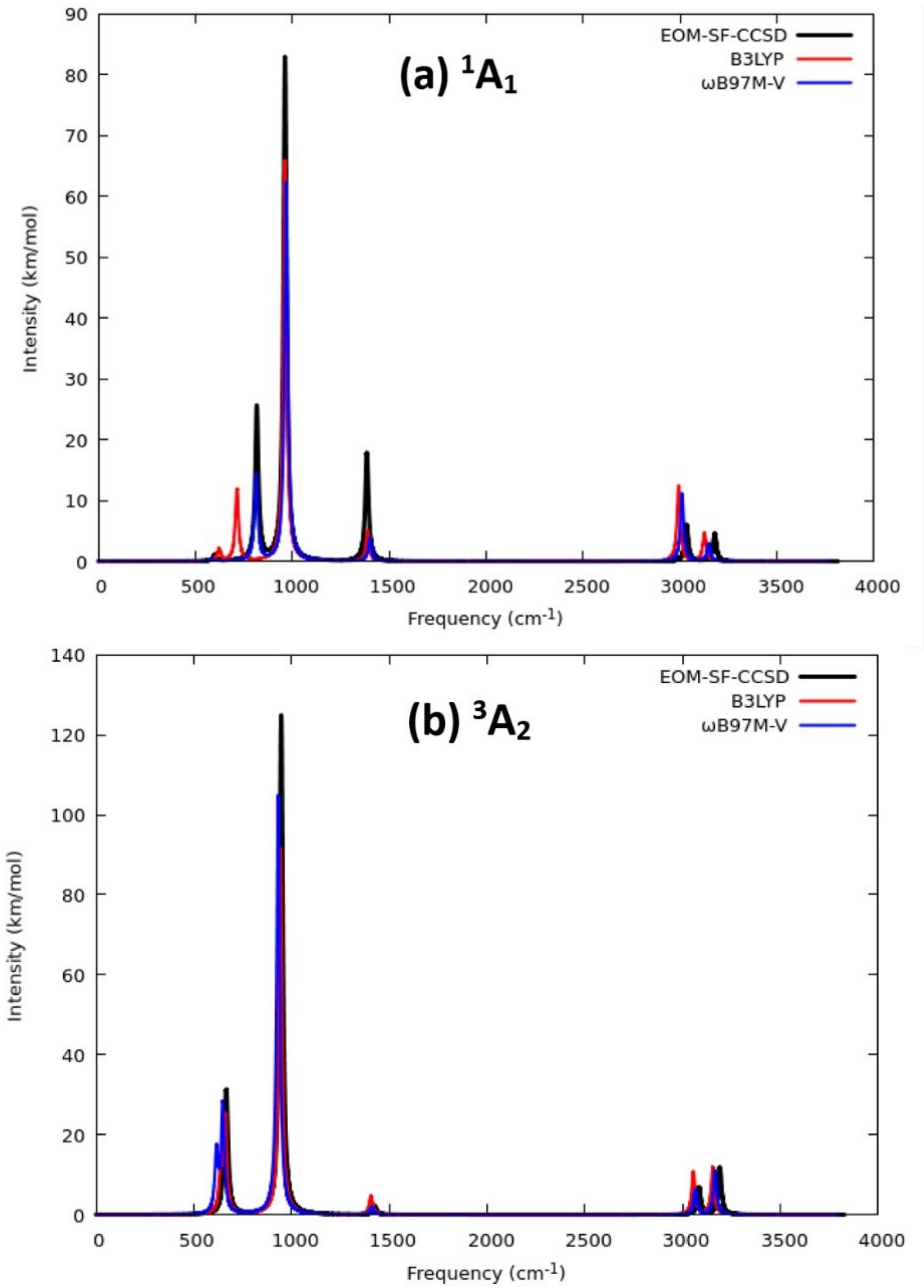

Figure S8: Comparison of computational methods' unscaled harmonic spectra for the lowest singlet and triplet states of $RhCH_2^+$. The full frequency range of fundamental transitions is depicted, even though the experimental spectra only cover $\leq$ 2100 cm^{-1}. All three methods used the same cc-pVTZ-PP basis set on the metal atom and cc-pVTZ basis on the C/H atoms, as well as the ECP28MDF core potential.

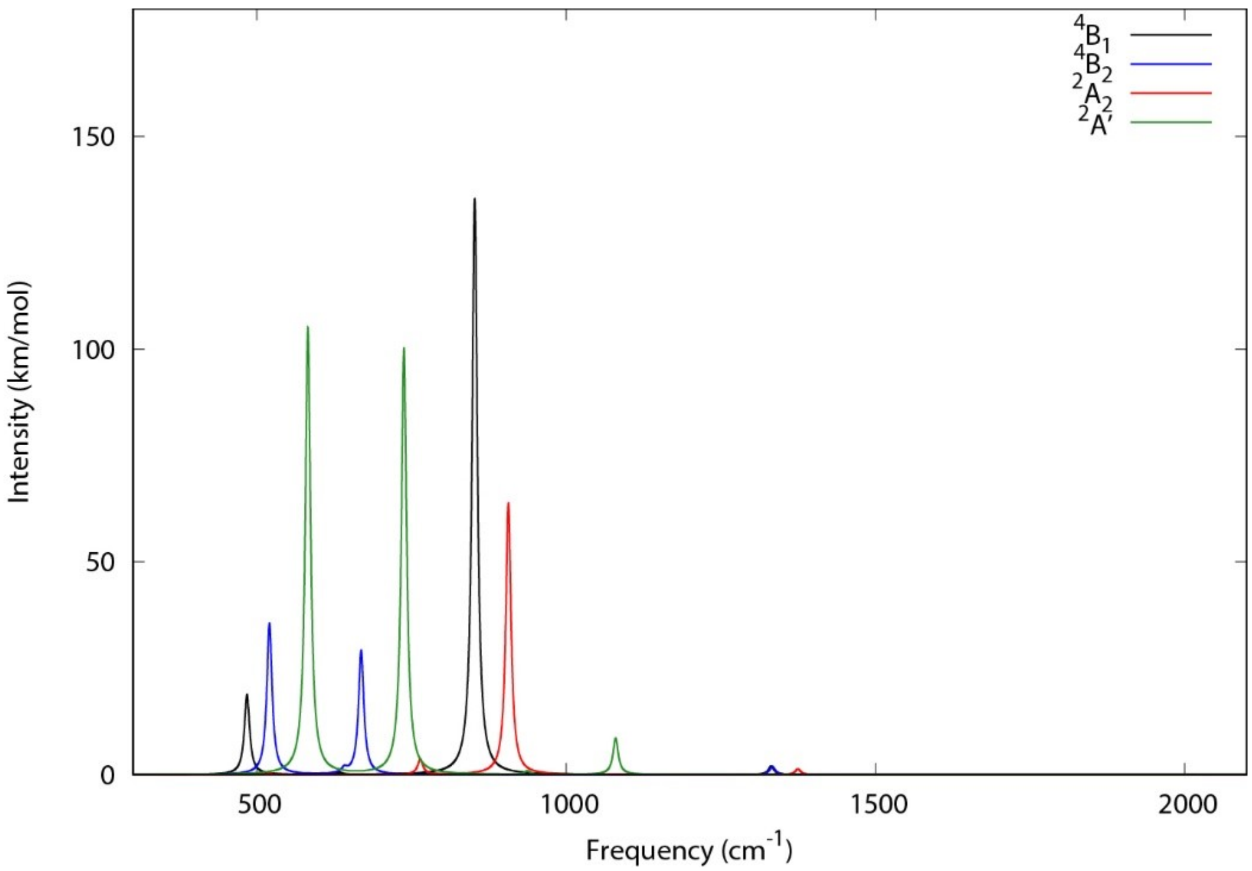

Figure S9: Unscaled harmonic spectra for the low-lying electronic states of $[Ru,C,2H]^+$, computed with ωB97M-V/cc-pVTZ-PP. The relative electronic energies of these configurations are 0.000 eV ($^4B_1$), +0.013 eV ($^4B_2$), +0.383 eV ($^2A_2$), and +0.606 eV ($^2A'$).

Table S1: Ground and excited electronic states for $[M,C,2H]^+$ isomers with electronic occupation for selected states calculated at the B3LYP/def2-TZVPPD and CCSD(T)/def2-TZVPPD//CCSD/def2-TZVPPD (in italics) levels. The relative energy at 0 K, E_{Rel}, is given with respect to the lowest energy isomer for the metal involved.

Species	State	E_{Rel} (eV)	E_{Rel} (eV) Lit.	Valence MO Occupation
$RuCH_2^+$	$^4B_2$	0.000 / *0.045*	0.412[a]	$(1a_1)^2(1b_1)^2(2a_1)^1(1a_2)^2(3a_1)^1(1b_2)^1$
	$^4B_1$	0.001 / *0.044*	0.416	$(1a_1)^2(1b_1)^2(2a_1)^2(1a_2)^1(3a_1)^1(1b_2)^1$
	$^4A_2$	0.145 / *0.091*	0.408	$(1a_1)^2(1b_1)^2(2a_1)^1(1a_2)^1(3a_1)^1(1b_2)^2$
	$^2A_2$	0.131 / *0.150*	0.000	$(1a_1)^2(1b_1)^2(2a_1)^2(1a_2)^1(3a_1)^0(1b_2)^2$
	$^2A_1$	0.157 / *0.134*	0.052	$(1a_1)^2(1b_1)^2(2a_1)^1(1a_2)^2(3a_1)^0(1b_2)^2$
	$^2B_2$	0.396		
	$^2B_2$	0.664	0.867	
	$^2B_1$	0.664		$(1a_1)^2(1b_1)^2(2a_1)^2(1a_2)^1(3a_1)^1(1b_2)^1$
	$^2B_2$	0.695		
	$^2A''$	0.938		
	$^6A'$	1.425		$(1a_1)^2(1b_1)^2(2a_1)^1(1a_2)^1(3a_1)^1(1b_2)^1(2b_1)^1$
$HRuCH^+$	$^2A'$	0.029 / *0.000*		
	$^2A''$	0.583		
	$^4A''$	1.318		
	$^4A'$	2.410		
	^6A	3.562		
	$^6A''$	3.611		
$(H_2)RuC^+$	$^2A'$	0.467		
	$^2A''$	0.800		
	$^4A''$	2.469		
	$^6B_1$	4.483		
$HHRuC^+$	$^2A'$	1.723		
	$^4B_1$	3.482		
	$^6A_1$	6.118		
$RhCH_2^+$	$^1A_1$ [b]	-0.020		$(1a_1)^2(1b_1)^2(2a_1)^2(1a_2)^2(3a_1)^0(1b_2)^2$
	$^1A_1$	0.000 / *0.000*	0.000[c]	$(1a_1)^2(1b_1)^2(2a_1)^2(1a_2)^2(3a_1)^0(1b_2)^2$
	$^1A_2$	0.651 / *0.567*		$(1a_1)^2(1b_1)^2(2a_1)^2(1a_2)^1(3a_1)^1(1b_2)^2$
	$^1A_1$	0.664		$(1a_1)^2(1b_1)^2(2a_1)^1(1a_2)^2(3a_1)^1(1b_2)^2$
	$^3A_2$	0.182 / *0.168*	0.195	$(1a_1)^2(1b_1)^2(2a_1)^2(1a_2)^1(3a_1)^1(1b_2)^2$
	$^3A_1$	0.196 / *0.179*	0.212	$(1a_1)^2(1b_1)^2(2a_1)^1(1a_2)^2(3a_1)^1(1b_2)^2$
	$^3B_2$	0.659	0.846	$(1a_1)^2(1b_1)^2(2a_1)^2(1a_2)^2(3a_1)^1(1b_2)^1$
	$^3B_1$	1.350	1.023	$(1a_1)^2(1b_1)^2(2a_1)^2(1a_2)^1(3a_1)^2(1b_2)^1$
	$^5A''$ [d]	1.785		$(1a_1)^2(1b_1)^2(2a_1)^1(1a_2)^2(3a_1)^1(1b_2)^1(2b_1)^1$
	$^5B_2$	2.259		
$HRhCH^+$	$^1A'$	0.827 / *0.762*		
	$^1A'$	2.561		
	$^1A''$	2.317		
	$^3A'$	1.531		

	$^3A''$	2.178
	$^5A'$	3.616
$(H_2)RhC^+$	$^1A'$	0.793
	$^3A'$	2.681
	$^5B_1$	4.729
	$^5A''$	5.031
$HHRhC^+$	$^3A''$	3.422
	$^1A_1$	4.037

[a] Carter, E. A.; Goddard, W. A. *J. Am. Chem. Soc.* **1986,** *108*, 2180-2191.

[b] This $RhCH_2^+$ ($^1A_1$) state is interpreted as an artefact, but included nevertheless.

[c] Musaev, D. G.; Koga, N.; Morokuma, K. *J. Phys. Chem.* **1993,** *97*, 4064-4075.

[d] This state is non-planar (∠HCRhH = 157°) but close enough that the orbitals can still be assigned using C_{2v} symmetry designations.
